# Cryo-EM structure of the respiratory I + III_2_ supercomplex from *Arabidopsis thaliana* at 2 Å resolution

**DOI:** 10.1038/s41477-022-01308-6

**Published:** 2022-12-30

**Authors:** Niklas Klusch, Maximilian Dreimann, Jennifer Senkler, Nils Rugen, Werner Kühlbrandt, Hans-Peter Braun

**Affiliations:** 1 Department of Structural Biology, Max-Planck-Institute of Biophysics, Frankfurt, Germany; 2 Institut für Pflanzengenetik, Leibniz Universität Hannover, Hannover, Germany

**Keywords:** Plant molecular biology, Cryoelectron microscopy

## Abstract

Protein complexes of the mitochondrial respiratory chain assemble into respiratory supercomplexes. Here we present the high-resolution electron cryo-microscopy structure of the *Arabidopsis* respiratory supercomplex consisting of complex I and a complex III dimer, with a total of 68 protein subunits and numerous bound cofactors. A complex I-ferredoxin, subunit B14.7 and P9, a newly defined subunit of plant complex I, mediate supercomplex formation. The component complexes stabilize one another, enabling new detailed insights into their structure. We describe (1) an interrupted aqueous passage for proton translocation in the membrane arm of complex I; (2) a new coenzyme A within the carbonic anhydrase module of plant complex I defining a second catalytic centre; and (3) the water structure at the proton exit pathway of complex III_2_ with a co-purified ubiquinone in the Q_O_ site. We propose that the main role of the plant supercomplex is to stabilize its components in the membrane.

## Abstract (web summary)

A high-resolution electron cryo-microscopy structure of the mitochondrial respiratory supercomplex I + III_2_ from *Arabidopsis thaliana* shows that the component complexes stabilize each other. A complex I-ferredoxin, subunit B14.7 and the newly defined P9 subunit mediate supercomplex formation.

## Main

The main electron transport pathway of the mitochondrial electron transfer chain (ETC) has three sections that each correspond to a separate membrane protein complex: (1) the NADH dehydrogenase complex, known as complex I, is the main entrance point for electrons into the ETC, where complex I catalyses electron transfer from NADH to ubiquinone; (2) cytochrome *c* reductase, often referred to as complex III, accepts ubiquinol from complex I and passes electrons to cytochrome *c*; and (3) cytochrome *c* oxidase, also known as complex IV, catalyses the transfer of electrons from cytochrome *c* to molecular oxygen. Together with complex II, which transfers electrons from succinate to ubiquinone, complexes I, III and IV were first identified in the inner membrane of mammalian mitochondria and originally assumed to exist as separate entities^1^. However, there was some biochemical evidence that the ETC complexes form larger assemblies in the membrane^2,3^. Defined ETC assemblies, referred to as respiratory supercomplexes or respirasomes, were first characterized by mild native polyacrylamide gel electrophoresis^4^. The main respiratory supercomplex of mammalian mitochondria consists of complex I, a complex III dimer (complex III_2_) and up to two monomers of complex IV. The structure of this respirasome has been determined by single-particle electron cryo-microscopy (cryo-EM)^5–7^ and electron cryo-tomography^8^. The ETC generates a proton gradient across the inner mitochondrial membrane, which is used by the ATP synthase to produce ATP^9^.

Apart from its main role in mitochondrial ATP production, the ETC of plants has additional functions, some of which are related to photosynthesis^10^. The plant ETC includes several alternative oxidoreductases, resulting in branched electron transfer pathways. Furthermore, the canonical complexes of the plant ETC comprise subunits absent in animals and fungi. For example, plant complex III_2_ includes the two subunits of the mitochondrial processing peptidase (MPP), which removes pre-sequences from nuclear-encoded mitochondrial proteins upon import^11^. In yeast and mammals, pre-protein processing is carried out by a soluble enzyme in the mitochondrial matrix^12^. Mitochondrial complex I of plants has a characteristic γ-type carbonic anhydrase (γCA) module^13^ that is thought to be involved in the re-use of mitochondrial CO_2_ for carbon assimilation in the chloroplasts^14^. The γCA module sits on the matrix side of the complex I membrane arm and has three γCAs. The structures of *Arabidopsis* and *Polytomella* complex I recently revealed a protein bridge between the γCA module and the complex I peripheral arm^15^ that includes an unusual ferredoxin (C1-FDX) and may stabilize the complex. Both the γCA and the bridge modules are absent in complex I from mammals and yeasts but were recently discovered in complex I from protozoa^16,17^, suggesting that they were complex I components of the last eukaryotic common ancestor.

The cryo-EM structures of plant complexes I, III_2_ and IV have recently been determined at resolutions ranging from 2.9 to 3.9 Å (refs. 15,18–20). As in mammals and fungi, the plant ETC complexes form supercomplexes^21,22^. However, the plant I + III_2_ + IV supercomplex is fragile, most likely due to the weak interaction of complexes III_2_ and IV (ref. 20). In contrast, the plant I + III_2_ supercomplex is stable and abundant^21,22^. Low-resolution structures of the supercomplex from *Arabidopsis* (18 Å) and potato (20 Å) have been obtained by negative-stain electron microscopy (EM)^23,24^, but high-resolution cryo-EM structures are required to understand the plant-specific features of the supercomplex, in particular the arrangement and functional interplay of the carbonic anhydrase and bridge modules of complex I and the MPP module of complex III.

In this Article, we present the high-resolution cryo-EM structure of the *Arabidopsis* I + III_2_ supercomplex. The structure offers new insights not only into the function of the individual supercomplex components in near-atomic detail, but also into supercomplex assembly. The interface between complex I and complex III_2_ is more extensive than in the mammalian I + III_2_ supercomplex^25^. Supercomplex formation changes the conformation of the complex I membrane arm. The change appears to be induced by the B14.7 kDa subunit, which was absent in the previously determined structures of free complex I from plants, and indirectly depends on C1-FDX.

## Results

### 2 Å structure of the *Arabidopsis* I + III_2_ supercomplex

**Fig. 1:**
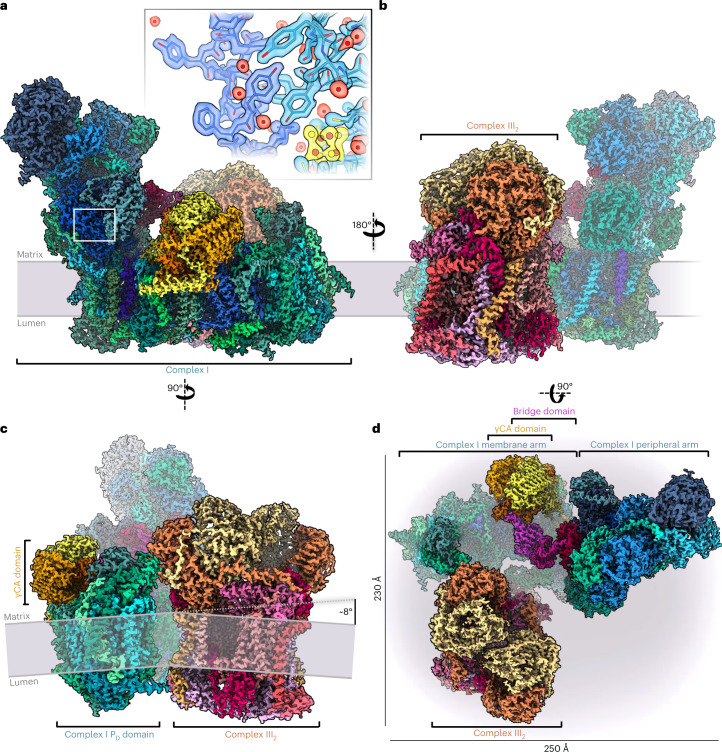
2 Å structure of the *Arabidopsis* I *+* III_2_ supercomplex. **a**–**c**, Views from the plane of the inner mitochondrial membrane (grey), with complex I in front (**a**), with complex III_2_ in front (**b**), and from the tip of the complex I membrane arm (**c**). The supercomplex protrudes into the mitochondrial matrix and the cristae lumen. The 14 complex I core subunits, which are conserved in bacterial and mitochondrial complex I, are drawn in shades of blue; accessory subunits in shades of green; the three subunits of the γCA module in yellow, orange and red; the three subunits of the bridge module in pink, purple and red. In **a**, the inset shows a typical map region near FeS cluster N2 (yellow), and water densities are red. In **c**, the inner mitochondrial membrane bends by ~8° around the supercomplex. **d**, Matrix view of the I + III_2_ supercomplex. Subunits protruding from the membrane are shown in strong colours. For detailed view of the high-resolution structure, see Supplementary Videos 1 and 2. For cryo-EM data processing and activity measurements, see Supplementary Figs. 2–5. All subunits are identified in Fig. 2. For comparison with the low-resolution map of the supercomplex obtained by single-particle negative-stain EM^23^, see Supplementary Fig. 6.

We determined the cryo-EM structure of the active *Arabidopsis* I + III_2_ supercomplex at 2 Å resolution (Fig. 1 and Supplementary Videos 1 and 2). The complex was isolated from digitonin-solubilized mitochondrial membranes and further purified in the synthetic digitonin analogue glyco-diosgenin (GDN) (Supplementary Fig. 1). Single-particle analysis resulted in a 2.36 Å reconstruction of the entire supercomplex (Supplementary Fig. 2). Particle subtraction and separate multibody refinement of the component complexes I and III_2_ improved the resolution to 2.03 Å, or 1.9 Å after density modification (Supplementary Fig. 3 and Supplementary Tables1–3). In addition, two slightly different conformations of the *Arabidopsis* I + III_2_ supercomplex were resolved by focused 3D classification with resolutions of up to 2.34 Å (Supplementary Figs. 2 and 4, and Supplementary Tables 1–3).

**Fig. 2:**
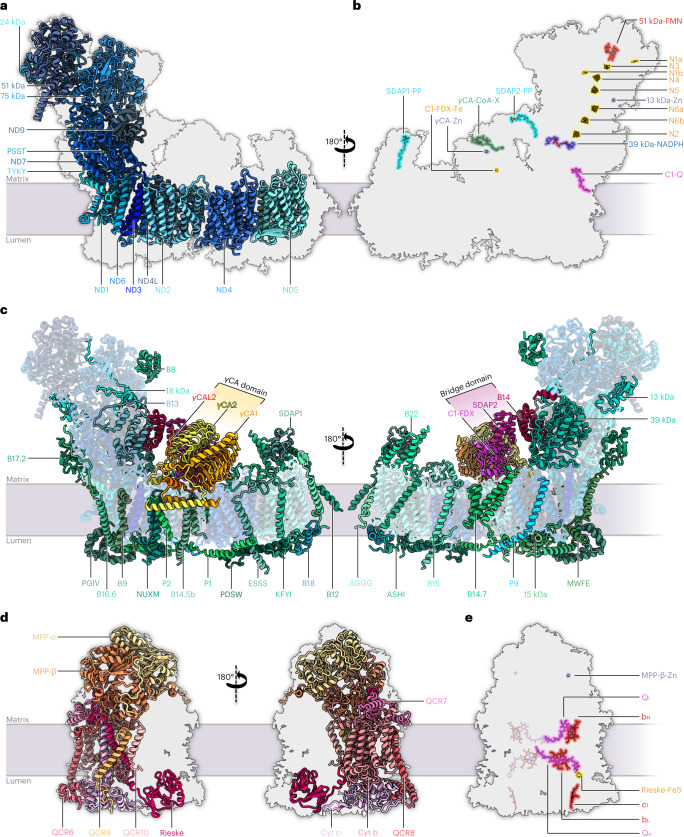
Subunit composition of complex I and complex III_2_ within the *Arabidopsis* I + III_2_ supercomplex. **a**–**c**, Atomic model of complex I, showing the 14 core subunits in shades of blue (**a**), cofactors bound to complex I (N, FeS clusters; Q, ubiquinone/ubiquinol; FMN, flavine mononucleotide) (**b**) and accessory subunits of mitochondrial complex I (**c**). Conserved accessory subunits are shown in shades of green, the subunits of the carbonic anhydrase (CA) domain in yellow and orange, and the subunits of the bridge domain in red and pink. The newly identified subunit P9 is light blue. Subunit nomenclature as for bovine complex I (ref. 80) except for non-conserved accessory subunits (for details, see Supplementary Table 5). **d**,**e**, Atomic model of complex III_2_, showing the structure of the ten subunits of one complex III monomer within the complex III dimer (for details, see Supplementary Table 6) from opposite directions (**d**) and bound cofactors of complex III_2_ (b_H_, b_L_, c_1_: haem groups attached to cytochrome b and c_1_; FeS: iron–sulphur cluster attached to the Rieske protein; Q_i_, Q_o_: quinone binding sites; Zn: zinc^2+^ bound to MPP-β) (**e**). For details and bound lipids, see Extended Data Fig. 2.

Purified *Arabidopsis* I + III_2_ supercomplex had a NADH:cytochrome *c* oxidoreduction activity of 2.5 U mg^−1^ (Supplementary Fig. 5), similar to the mammalian I + III_2_ supercomplex^25^. Mass spectrometry (MS) identified 48 different subunits of complex I and 10 different subunits of complex III, some of which were present as pairs of isoforms (Supplementary Table 4). The cryo-EM map reveals the general architecture of the supercomplex (Fig. 1) and the arrangement of its subunits within it (Fig. 2, Table 1 and Supplementary Tables 5 and 6; for calculated molecular masses, isoelectric points and hydrophobicity of the subunits, see Supplementary Tables 7–9). The structure of complex III_2_ in our map agrees closely with that of mung bean complex III_2_ (ref. 20). Furthermore, the structure of complex I in the supercomplex generally agrees with that of the free, unassociated complex^15^, with the following three exceptions: (1) We identified a copy of subunit B14.7, which was not found in the previous cryo-EM maps of plant complex I^15,18,19^. In the supercomplex, this subunit sits at the interface of complexes I and III_2_ at one of their three interaction sites. (2) Subunit MNLL^15^ was re-assigned as NUXM because it is clearly homologous to the fungal complex I subunit NUXM rather than mammalian MNLL^26^. (3) The high-resolution map density allowed us to determine the sequence of a plant-specific subunit next to the ubiquinone binding site in the membrane arm^15^, which is encoded by the genetic locus At1g67785 (TAIR; https://www.arabidopsis.org/). We propose that this novel complex I subunit should be referred to as P9, as the other plant- and green algae-specific complex I subunits are known as P1 to P8 (refs. 15, 27, 28).

**Table 1:** Subunits of the *Arabidopsis* I + III_2_ supercomplex as shown in Fig. 2 and Supplementary Table 4

Complex I, peripheral arm (including bridge domain), 17 subunits			
Core subunits (7)	Conserved accessory subunits (9)		Other accessory subunit (1)
24 kDa	13 kDa	B17.2	C1-FDX
51 kDa	18 kDa	SDAP-2	
75 kDa	39 kDa		
TYKY-1	B8		
PSST	B13		
ND7	B14		
ND9	B14.5a		

Complex I, membrane arm (including carbonic anhydrase domain), 31 subunits			
Core subunits (7)	Conserved accessory subunits (18)		Other accessory subunits (6)
ND1	15 kDa	B18	CA1/CA3
ND2	AGGG	B22	CA2
ND3	ASHI	ESSS-1	CAL2/CAL1
ND4	B9	KFYI	P1 (SGDH)
ND4L	B12-2	NUXM	P2
ND5	B14.5b	MWFE	P9
ND6	B14.7	PDSW-1	
	B15	PGIV-2	
	B16.6-2	SDAP-1	

Complex III, 10 subunits (two copies each in the dimeric complex III)			
Core subunits (3)	Conserved accessory subunits (5)		Other accessory subunits (2)
Cyt b	QCR6-1	QCR9	MPP-α-1
Cyt c_1_-1	QCR7-2	QCR10	MPP-β
Rieske-1	QCR8-1		

Core subunits are the minimal set necessary for complex I and complex III_2_ function and are conserved in prokaryotes and mitochondria. Accessory subunits are additional proteins forming part of the two complexes in mitochondria. They can be divided into conserved and non-conserved accessory subunits. Extensions −1/−2 indicate isoforms of *Arabidopsis* complex I and complex III_2_ subunits. The dominant isoform was fitted to the map and is shown in the structures. Subunit B14.5a was found in purified *Arabidopsis* I + III_2_ supercomplex by MS (Supplementary Table 4) but not identified in the map. For protein nomenclature and accession numbers, see Supplementary Tables 5 and 6

**Extended Data Fig. 1:**
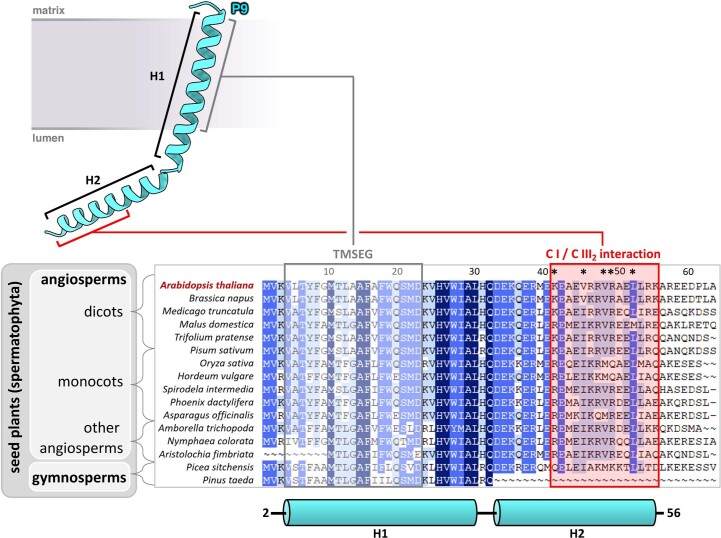
Phylogenetic analysis of complex I subunit P9. P9 is a two-helix (H1, H2) peptide that spans the inner mitochondrial membrane from the matrix to the lumenal side by its transmembrane segment (TMSEG). For phylogenetic analysis, *Arabidopsis* P9 (At1g67785) was used to search the non-redundant protein sequences (nr) database at the National Center for Biotechnology Information (NCBI, “https://www.ncbi.nlm.nih.gov/”) using standard settings. >100 homologous sequences were identified below an E-value of 1e-4. All sequences were from seed plants (spermatophyta). Sixteen sequences were selected for building a multiple sequence alignment using Clustal Omega (https://www.ebi.ac.uk/Tools/msa/clustalo/); five sequences for dicots and monocots, respectively, and all identified sequences of other spermatophyta clades. Amino acid positions conserved in 16/16 sequences are highlighted in dark blue, amino acid positions conserved in ≥14/16 sequences in mid-blue and amino acid positions conserved in ≥10/16 sequences in light blue. Phylogenetic clades are indicated to the left of the alignment. The topology model of P9 is indicated above the alignment. The model was built into the cryo-EM density of the *Arabidopsis* I + III_2_ supercomplex from valine at position 2 to alanine at position 56. The positions of its two alpha-helices are shown below the alignment. The complex III_2_ interacting segment is shown in red. Residues K41, V45, V48, R49, L52 of *Arabidopsis* P9 that interact directly with complex III_2_ are indicated by asterisks (see Fig. 4c and d). Accession of sequences. *Arabidopsis thaliana*: At1g67785, *Brassica napus*: CAF2186799, *Medicago truncatula*: XP_013446710, *Malus domestica*: XP_028959763, *Trifolium pratense*: XP_045824609, *Pisum sativum*: XP_050898094, *Oryza sativa*: NP_001392381, *Hordeum vulgare*: XP_044978029, *Spirodela intermedia*: CAA2619857, *Phoenix dactylifera*: XP_008797106, *Asparagus officinalis*: XP_020253321, *Amborella trichopoda*: XP_020527559, *Nymphaea colorata*: XP_031475842, *Aristolochia fimbriata*: KAG9445531, *Picea sitchensis*: ABK24944, *Pinus taeda*: AFG45619.

Earlier, P9 was identified in plant complex I by biochemical experiments^29,30^. On the basis of low sequence similarity, this subunit was suggested to possibly correspond to mammalian SGDH^31^, but our high-resolution cryo-EM structure now excludes this possibility. Phylogenetic analysis revealed that P9 homologues are present only in seed plants (Extended Data Fig. 1). However, the cryo-EM structure of complex I from the green alga *Polytomella* includes an unknown subunit exactly at the position of P9 in *Arabidopsis* complex I (ref. 15). As P9 is small and partially hydrophobic, homologues might have escaped detection due to limited sequence similarity. In contrast, complex I structures from animals, fungi and *Tetrahymena* lack a subunit at the P9 position.

**Extended Data Fig. 2:**
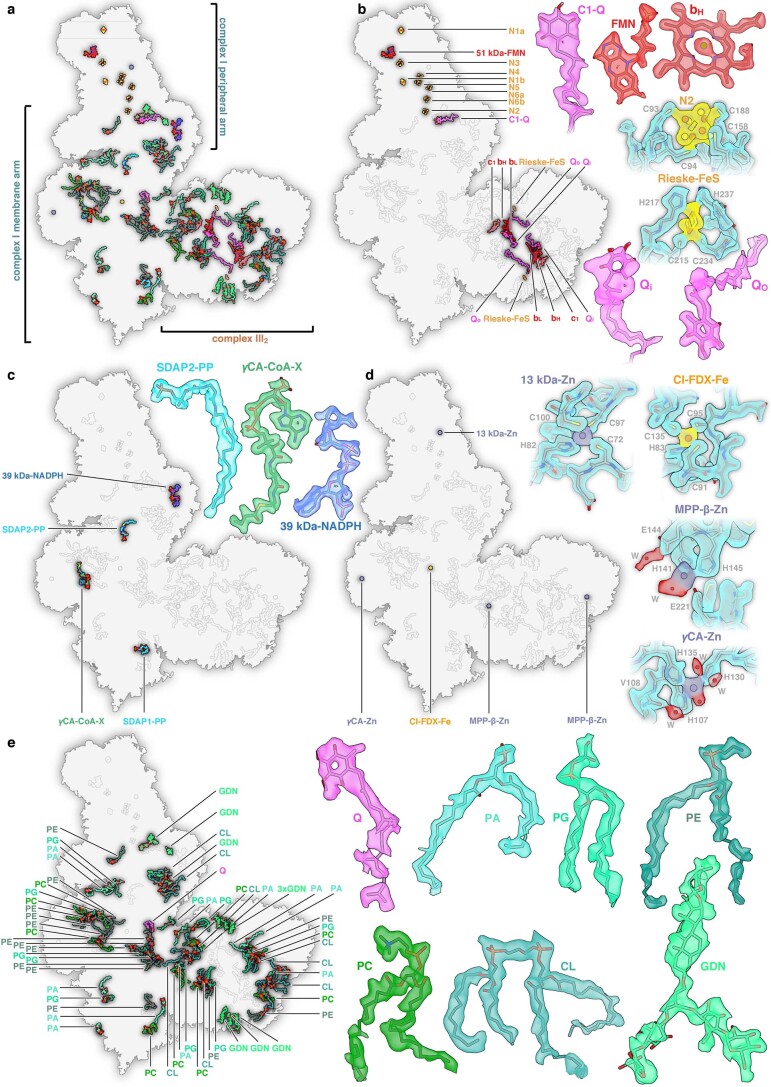
Bound cofactors, metal ions and lipids in the *Arabidopsis* I + III_2_ supercomplex. **a**, Overview from the matrix side. **b**, Cofactors involved in respiratory electron transport. Complex I: FMN (Flavin mononucleotide), N1a, N1b, N2, N3, N4, N5, N6a, N6b (8 FeS clusters) and C1-Q (ubiquinol/ubiquinone at the Q reduction site). Complex III_2_: two copies each of heme b_H_, b_L_ and c_1_, Rieske FeS, plus ubiquinone/ubiquinol at the Q_o_ and Q_i_ sites. **c**, Other cofactors: PP (S-acyl-4ʼ-phosphopantetheine bound to SDAP1 and SDAP2), CoA-X (Butyryl-or Crotonyl-Coenzym A bound to γCA), and NADPH bound to the 39 kDA subunit. **d**, Bound metal ions. **e**, Lipids and detergent molecules, with numbers of copies in round brackets. PA, phosphatidic acid (11); PC, phosphatidylcholine (9); PE, phosphatidylethanolamine (14); PG, phosphatidylglycerol (10); CL, cardiolipin (8); GDN, glyco-diosgenin (10); Q, ubiquinone/ubiquinol (not bound in the Q reduction site of complex I or in the Q_o_ and Q_i_ sites of complex III_2_). Densities and models of selected cofactors, metal ions and lipids are highlighted.

Apart from high-resolution structures of the polypeptides, our density map of the I + III_2_ supercomplex contains a total of 94 bound cofactors, lipids and metal ions (Fig. 2b,e and Extended Data Fig. 2), including a butyryl/crotonyl-CoA molecule in the γCA module of complex I and ubiquinone/ubiquinol (Q) in the two Q_O_ sites of the complex III dimer. Furthermore, we modelled 4,837 water molecules.

### Subunit B14.7 increases the membrane arm curvature of *Arabidopsis* complex I

**Fig. 3:**
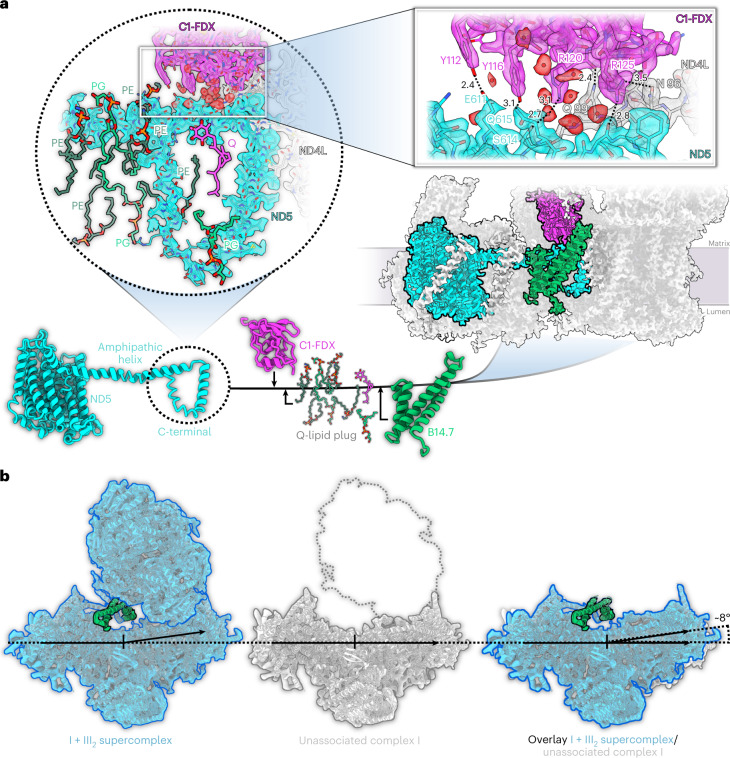
The role of subunit B14.7 and C1-FDX in I + III_2_ supercomplex formation. **a**, Subunit B14.7 (green) and C1-FDX (magenta) interact closely with the C-terminal loop of ND5 (cyan). The loop is surrounded by a set of lipids and a Q molecule (circled inset). C1-FDX sits on top of the amphipathic helix of ND5 and stabilizes it via a tight hydrogen bond network (black dotted lines) including well-defined water molecules (square inset; all distances in Å). **b**, Supercomplex (left, blue) with subunit B14.7 (green) compared with the structure of unassociated complex I (centre; grey^15^), which does not have the B14.7 subunit. The position of the complex III dimer in the supercomplex is indicated by a grey dotted outline. An overlay (right) indicates that in the supercomplex the membrane arm of complex I rotates towards complex III_2_ by ~8°, resulting in a more extensive contact surface, which would stabilize the supercomplex. PE, phosphatidylethanolamine; PG, phosphatidylglycerol; Q, ubiquinone/ubiquinol.

Complex III_2_ binds to the inner curved surface of the complex I membrane arm (Fig. 1). This overall architecture is well conserved between mammals, yeasts and plants^8^. In ovine mitochondria, the B14.7 subunit is located at the interface of complexes I and III_2_ (ref. 25). In plants, this subunit is easily lost during purification of unassociated complex I and is absent in the plant complex I structures reported so far^15,18,19^. In the *Arabidopsis* I + III_2_ supercomplex, B14.7 is present (Fig. 3). Together with subunit C1-FDX and a set of lipids (five phosphatidylethanolamine (PE), three phosphatidylglycerol (PG) and one Q), B14.7 binds tightly to the C-terminal loop of ND5 at the centre of the membrane arm close to the complex III_2_ interface (Fig. 3a). The B14.7/C1-FDX/lipid arrangement stabilizes this loop together with an amphipathic helix that runs towards the main ND5 transmembrane domain at the tip of the membrane arm. This tight interaction increases the curvature of the membrane arm, enabling close contacts to the complex III dimer (Fig. 3b), stabilizing the supercomplex.

### A newly defined plant-specific subunit at the interface of complexes I and III_2_

**Fig. 4:**
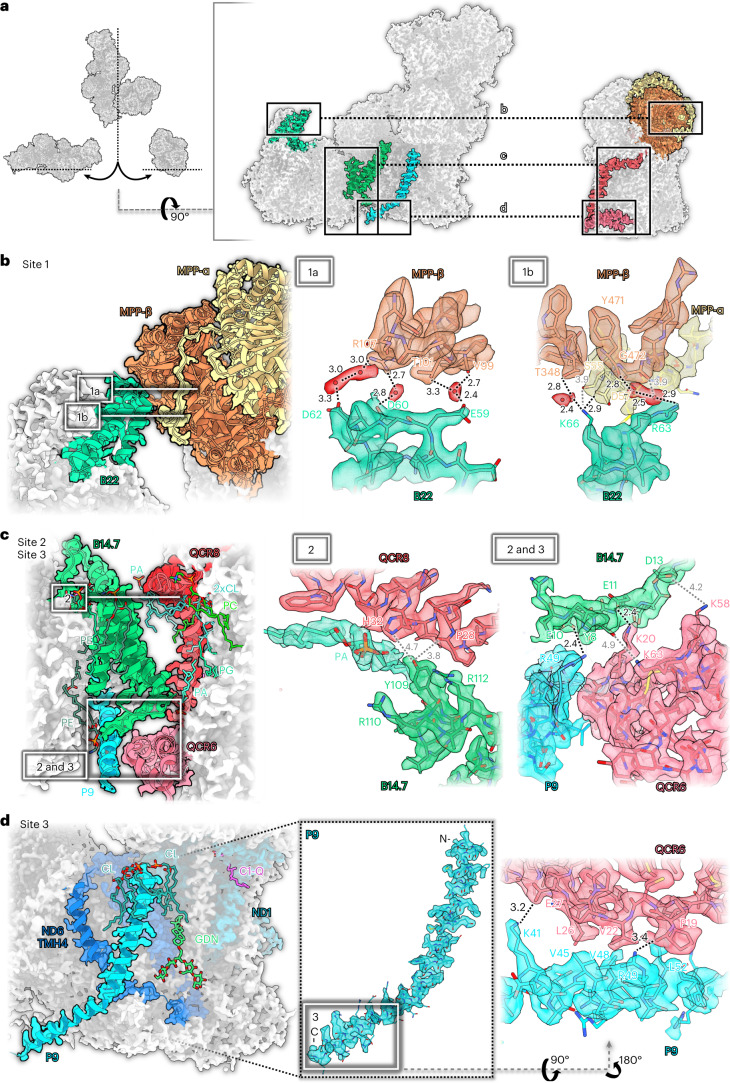
Three interaction sites of complexes I and III_2_ in the *Arabidopsis* supercomplex. **a**, Overview. **b**, Interaction site 1: B22 (green) of complex I binds to MPP-β (orange) and MPP-α (yellow) of complex III_2_. 1a and 1b show two roughly orthogonal views of interaction site 1. Interaction is mediated by hydrogen bonds including water molecules (red) and salt bridges. **c**, In site 2, B14.7 of complex I binds to QCR8 (red) and QCR6 (pink) of complex III_2_. In addition to polar contacts, the interaction is mediated by a set of membrane phospholipids. In site 3, binding to QCR6 involves the newly identified subunit P9 (light blue) of complex I. **d**, Also in site 3, the C-terminal part of P9 binds to QCR6 of complex III_2_ by salt bridges and hydrophobic contacts. Interacting amino acid residues are indicated by the one-letter code. Lipids: CL, cardiolipin; GDN, synthetic digitonin analogue glyco-diosgenin; PA, phosphatidic acid; PC, phosphatidylcholine; PE, phosphatidylethanolamine; PG, phosphatidyl-glycerol; Q, ubiquinone/ubiquinol. The network of hydrogen bonds (in Å) is indicated by black (<3.5 Å) or grey (>3.5 Å) dotted lines.

In the *Arabidopsis* supercomplex, complexes I and III_2_ interact at three distinct sites (Fig. 4). Sites 1 and 2 are partially conserved between *Arabidopsis* and the ovine I + III_2_ supercomplex. By contrast, site 3 involves the plant-specific subunit P9 and therefore appears to be unique to plants. Interactions at all three sites are based on several hydrogen bonds, salt bridges and van-der-Waals contacts and include numerous lipids and water molecules (Fig. 4). Site 1 involves subunit B22 on the complex I side, which binds to the pre-protein-processing enzyme subunits MPP-β and MPP-α of complex III_2_ (Fig. 4b). In the ovine I + III_2_ supercomplex, B22 binds only to UQCRC1, which is homologous to MPP-β of plants, but has no protein-processing activity^12^. In the ovine I + III_2_ supercomplex, subunit B15 contributes to the interaction at this site^25^. Site 2 marks the point where the complex I subunit B14.7 interacts with subunits QCR8 and QCR6 of complex III_2_ (whereas in the ovine complex, B14.7 interacts with QCR7 rather than QCR6 (ref. 25). Subunits B14.7 and QCR8 are both flanked by well-defined lipids (Fig. 4c). Finally, site 3, which is absent from the ovine complex, involves the plant-specific subunit P9 of complex I and QCR6 of complex III_2_. P9 is a small, 62-residue subunit that spans the inner mitochondrial membrane once near the ubiquinone binding site. Its C-terminus protrudes from the membrane into the cristae lumen, sometimes referred to as the intercristal space, where it interacts tightly with QCR6 (Fig. 4d and Extended Data Fig. 1). Notably, P9 is situated next to transmembrane helix 4 (TMH4) of ND6, which is thought to change position in the transition between the active and deactive states of mammalian complex I (ref. 32). Judging from our structures, this movement cannot take place in the *Arabidopsis* I + III_2_ supercomplex because P9 locks ND6-TMH4 in its position (Fig. 4d).

**Extended Data Fig. 3:**
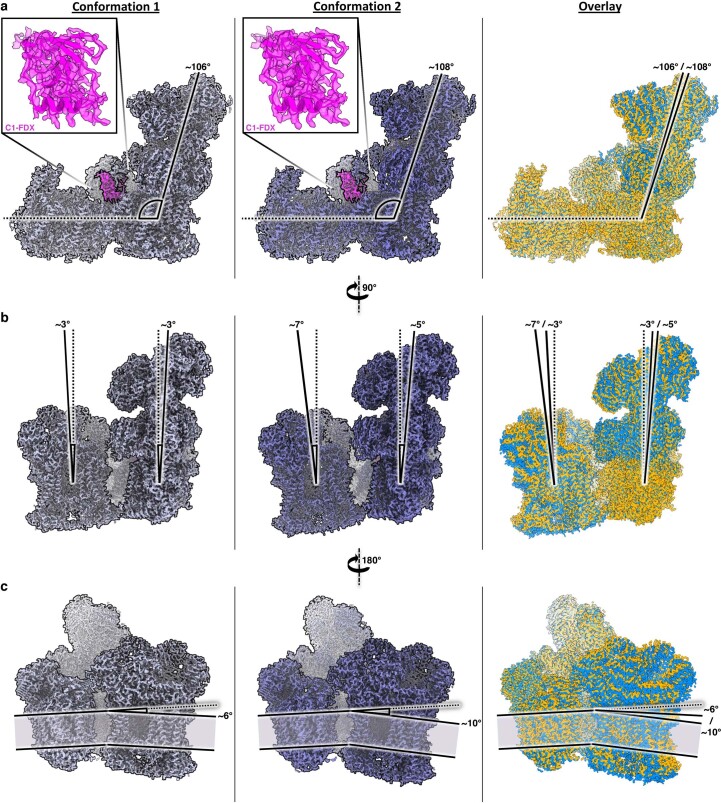
Conformations of the *Arabidopsis* I + III_2_ supercomplex. Confromation 1 and 2 with their corresponding overlays on the right. **a**, View from the plane of the inner mitochondrial membrane indicating the angle between the peripheral and membrane arm of complex I in the two resolved, slightly different conformations of the I + III_2_ supercomplex. The angle is 106° in conformation 1 and 108° in confromation 2. The ferredoxin subunit of the bridge domain (C1-FDX) is present and well-defined in both conformations (magenta insets; complex III_2_ removed for clarity). **b**, An end-on view of the supercomplex shows that the peripheral arm tilts by 3 to 5° and C III_2_ by 3 to 7 ° relative to the membrane normal (dotted lines). **c**, A view from the opposite direction reveals that the membrane plane of complex III_2_ tilts by 6° or 10° relative to the membrane plane of complex I (dotted line). The panels on the right show conformation 1 in orange and conformation 2 in blue. The inner mitochondrial membrane is shown in grey. The two confromations were obtained by focused 3D classification (Supplementary Figs. 2 and 4; Supplementary Tables 1 and 2).

**Extended Data Fig. 4:**
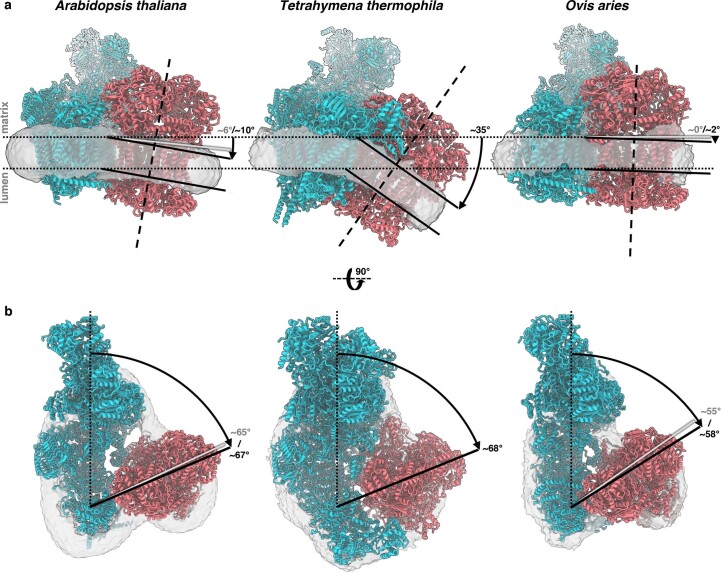
Conformations of the I + III_2_ supercomplex in different species. Comparison of I + III_2_ supercomplex from *Arabidopsis*, *Tetrahymena* and ovine mitochondria. Atomic models of complex I are blue, complex III_2_ is red. The position of the inner mitochondrial membrane is indicated by the detergent micelles in the cryo-EM maps lowpass-filtered to 7 Å (grey). Left: conformation 2 of *Arabidopsis thaliana*; centre: consensus refinement of *Tetrahymena thermophila;* right: open conformation of *Ovis aries*. **a**, View from the tip of the complex I membrane arm, indicating the angle to which the membrane around complex III_2_ bends relative to complex I. The different angles in conformation 1 in *Arabidopsis* and the closed conformation of the ovine supercomplex are indicated by grey lines. **b**, Angle included between complex III_2_ and the complex I membrane arm as seen from the mitochondrial matrix. The dotted line indicates the approximate long axis of the complex I membrane; the straight black line is the transverse axis of complex III_2_. Grey lines indicate the transverse axis of *Arabidopsis* conformation 1 and the ovine closed conformation. The *Arabidopsis* structure is from this study, the structure of the open *Ovis aries* supercomplex is from^25^ (PDB: 6QC3, EMDB: 4495) and that of the *Tetrahymena* supercomplex from^16^ (PDB: 7TGH, EMDB: 25882).

As seen from the matrix side, the consensus refinement structure as well as the two resolved I + III_2_ supercomplex conformations include an angle of 65–67° between the complex III dimer and the membrane arm of complex I (Extended Data Figs. 3 and 4). In the ovine supercomplex, the corresponding angle is ~55–58°. P9 increases this angle as it occupies the space between the two complexes. In *Tetrahymena*, even though it lacks P9, the angle is similar to *Arabidopsis* due to additional accessory subunits that prevent a tighter approach of complex III_2_ to the membrane arm of complex I.

In the composite structure of the *Arabidopsis* I + III_2_ supercomplex, the plane of the inner mitochondrial membrane is bent by about 8° (Fig. 1). In the two slightly different conformations of the supercomplex, the angular range is 6–10°. This indicates a slightly flexible arrangement of the two subcomplexes and raises the question whether the supercomplex actively induces membrane curvature or whether it adapts to a locally non-planar lipid bilayer. Although the lamellar cristae of plant mitochondria are known to be predominantly flat^33^, a minor local deviation from planarity would be difficult to detect (Extended Data Figs. 3 and 4).

### Details of the γCA catalytic sites

**Fig. 5:**
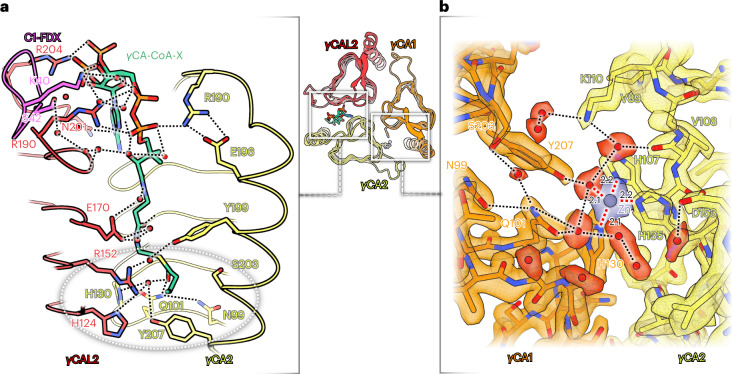
Catalytic sites in the γCA domain. Overview of the γCA domain (centre) with γCA1 (orange), γCA2 (yellow) and γCAL2 (red). **a**, Catalytic site at the γCA2–γCAL2 interface with bound butyryl- or crotonyl-CoA, here referred to as γCA-CoA-X (green). Subunits γCA2 and γCAL2 interact with the phosphate groups and the adenine ring of CoA-X either directly or across a chain (black dotted lines) of water molecules (red). The 3′ phosphate group adopts two alternative conformations, as indicated by two strong densities in the cryo-EM map (Extended Data Fig. 5). The butyryl or crotonyl group of CoA-X is located at the putative catalytic site of the γCAL2–γCA2 interface (grey dotted ellipse). **b**, Map density and fitted atomic model of the catalytic site at the γCA1–γCA2 interface. A zinc ion is coordinated by three histidines and one water molecule in tetrahedral geometry (red dotted lines). The active site is surrounded by a network of hydrogen bonds. For details, see Extended Data Figs. 5 and 6.

**Extended Data Fig. 5:**
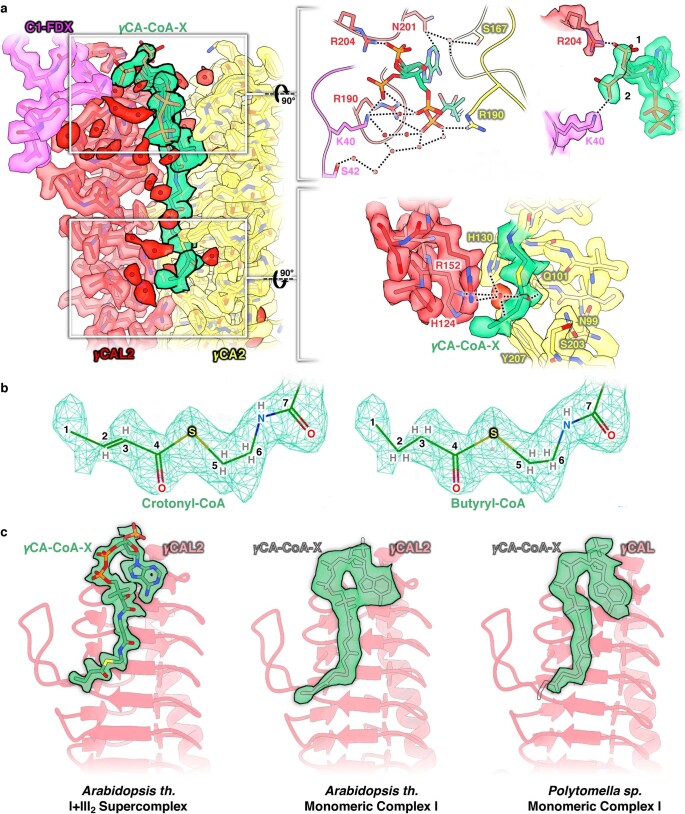
Butyryl- or Crotonyl-CoA at the γCAL2/γCA2 interface in the *Arabidopsis* I + III_2_ supercomplex. **a**, Left: Overview of atomic model and cryo-EM density at the complex I/III_2_ interface. γCAL2, light red; γCA2, yellow; C1-FDX, pink; water molecules, red densities or spheres; Butyryl- or Crotonyl-CoA (γCA-CoA-X), green. **a**, Right: Details of the CoA-X 3′-phosphate ADP binding region (top). The 3′-phosphate group can adopt two conformations as indicated by two strong densities in the cryo-EM map and interacts either with K40 of C1-FDX and R190 of γCAL2, or with R204 of γCAL2. γCAL2 N201 interacts either directly with the adenine ring or via a water molecule together with γCA2 S167. γCA2 R190 and a network of water molecules connect the diphosphate group of CoA-X to the three subunits at the interface. The butyryl or crotonyl group of CoA-X is located at the catalytic site of the γCAL2/γCA2 interface (below). The CoA-X oxygen of the C4 carbonyl group interacts via a water molecule with γCAL2 R152, γCAL2 H124, γCA2 H130 and γCA2 Y207. Q101 of subunit γCA2 is in direct hydrogen bond contact to the carbonyl group. Hydrogen bonds are indicated by dotted lines. **b**, Butyryl- or crotonyl-CoA fit the map density equally well. The model was drawn in Coot with the cryo-EM density at a contour level of 11.5 rmsd. **c**, A CoA-X density is also visible in the γCA domain of free complex I from *Arabidopsis* and *Polytomella*, but was not unambiguously identified at the lower map resolution^15^.

The γCA domain of *Arabidopsis* is a heterotrimer of two γCA subunits (γCA1 and γCA2; γCA1 can be replaced by the isoform γCA3) and one CAL2 subunit (CAL2 can be replaced by its isoform CAL1) (ref. 15). As in the homologous bacterial γCAs, which are homotrimers, three catalytic sites are located at the three subunit interfaces^34^. In bacteria, each of the three sites is active, as indicated by a bound metal ion coordinated by a set of three conserved histidines each. In the heterotrimeric γCA domain of *Arabidopsis*, only the catalytic site at the γCA1–γCA2 interface binds a metal ion^15^. The histidine sets at the γCA1–γCAL2 and γCA2–γCAL2 interface are incomplete and consequently cannot coordinate a metal ion. In the high-resolution structure of the *Arabidopsis* I + III_2_ supercomplex, we now find a coenzyme A at the potential catalytic site of the γCA2–γCAL2 interface (Fig. 5a and Extended Data Fig. 5). The density of this cofactor was visible in the structures of unassociated *Arabidopsis* and *Polytomella* complex I (ref. 15), but not interpreted due to insufficient resolution (Extended Data Fig. 5). Our present 2 Å map indicates that the cofactor is a butyryl- or crotonyl-CoA; the only difference between them is one single or double bond (Extended Data Fig. 5), which is not discernible. We therefore refer to this factor as γCA-CoA-X. The 3′-phosphate ADP and the diphosphate group of CoA-X interact with hydrophilic sidechains of γCA2, γCAL2 and C1-FDX either directly or via hydrogen bonds. The crotonyl or butyryl group of CoA-X is located at the catalytic site of the γCA2–γCAL2 interface, interacting via a central water molecule with two histidines and additional hydrophilic sidechains (Extended Data Fig. 5). Crotonyl-CoA is known to bind CO_2_ and can be carboxylated to (2*S*)-ethylmalonyl-CoA^35^. We assume that the catalytic site at the γCA2–γCAL2 interface is active and that crotonyl-CoA participates in the assimilation of CO_2_ for HCO_3_^−^ formation. CO_2_ binding at crotonyl-CoA would take place exactly at the position of the CO_2_ molecule in the canonical active site of γCA during bicarbonate conversion. Crotonyl-CoA is formed in plant mitochondria during catabolism of lysine and possibly tryptophan^36^. It is further formed during mitochondrial fatty acid biosynthesis.

**Extended Data Fig. 6:**
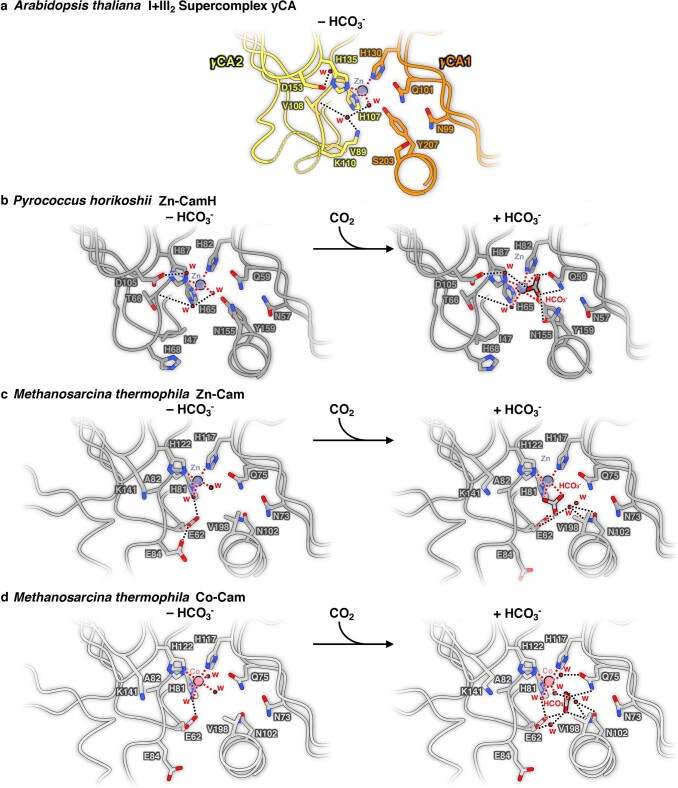
Active site architecture of gamma-type carbonic anhydrases. **a**, γCA-domain of complex I from *Arabidopsis*. The active site is located at the interface between the γCA2 (yellow) and γCA1 (orange) subunit. The three conserved histidines (H130 of γCA1, H107 and H135 of γCA2) coordinate a zinc ion (Zn) together with one water molecule (w) in a tetrahedral geometry (metal coordination shown by red dotted lines). The water molecule forms a hydrogen bond (black dotted lines) with an additional water that interacts with the polypeptide backbone of γCA2 V108 and residue γCA2 K110. A third water is coordinated by γCA2 D153. There appears to be no bound HCO_3_^−^. Coordination of Zn, the hydrogen network and conserved amino acids including Q101, N99, Y207 and S203 resemble the active Zn-CamH site of *Pyrococcus horikoshii*. **b**, *Pyrococcus horikoshii* Zn-CamH. Left: without HCO_3_^−^ (PDB: 1V3W), right: with HCO_3_^−^ (PDB: 1V67). **c**, *Methanosarcina thermophila* Zn-Cam. Left: without HCO_3_^−^ (PDB: 1QRG), right: with HCO_3_^−^ (PDB: 1QRL). Note that the zinc ion at the Cam site is coordinated by two further water molecules compared to the γCA2/γCA1 site in *Arabidopsis* and CamH. In addition, E62 and E84 that are known to be important for proton release replace V89 and K110 of γCA2 and I47 and H68 of CamH. **d**, *Methanosarcina thermophila* Co-Cam. Left: without HCO_3_^−^ (PDB: 1QQ0), right: with HCO_3_^−^ (PDB: 1QRE). A cobalt ion (Co) is coordinated by the three conserved histidines and three additional water molecules. In contrast to the heterotrimeric CA domain of *Arabidopsis* complex I, the bacterial Cam/CamH enzymes are homotrimers with three identical active sites located at the three subunit interfaces.

Our structure of the I + III_2_ supercomplex provides new insights into the architecture of the complete catalytic site at the γCA1–γCA2 interface. The higher resolution compared with the structure of the unassociated *Arabidopsis* complex I (ref. 15) now indicates the positions of water molecules. Apart from the three histidines, the central metal ion is coordinated by a water molecule in a tetrahedral geometry. This observation concurs with the catalytic site of the CamH γCA subclass that contains a central zinc ion and is known to be active^37^. Moreover, the network of hydrogen bonds and sidechains of the γCA1–γCA2 catalytic site resembles the network of CamH and differs from the one found in the Cam subclass where a zinc or cobalt ion is coordinated by two or three additional water molecules^34,38^. As a result, we can now clearly assign the γCA of plant complex I to the CamH subclass and the metal at the active site must be zinc (Fig. 5b and Extended Data Fig. 6). We conclude that the *Arabidopsis* complex I γCA1–γCA2 site is active.

### The aqueous passage in the membrane arm of *Arabidopsis* complex I

**Fig. 6:**
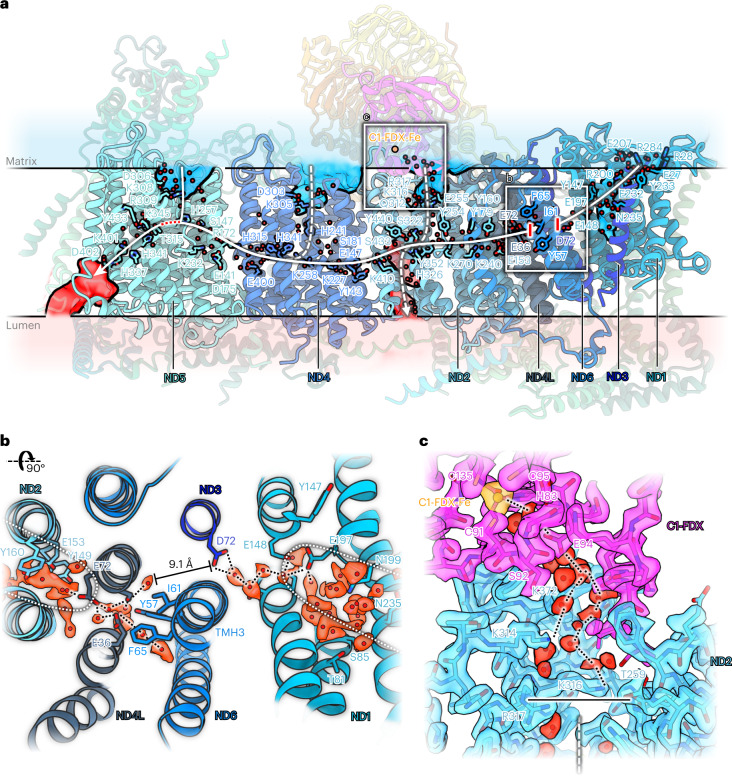
Proton transfer path in the membrane arm of *Arabidopsis* complex I. **a**, Conserved amino acids and water molecules (red spheres) in the membrane arm mark the central aqueous passage (white continuous line) and potential water channels (white dashed lines) to the mitochondrial matrix (transparent blue) or the cristae lumen (transparent red). The passage is interrupted at transmembrane helix 3 of subunit ND6 (red bars; see **b**). In subunit ND2, two potential half-channels connect the central aqueous passage to the matrix or the lumen. C1-FDX with its bound Fe ion sits next to the entrance of the ND2 matrix half-channel. The matrix and lumenal half-channel of ND5 are open. Proton leakage is prevented by a ~6 Å gap between ND5 H257 and T315 of the aqueous passage (red dotted line). **b**, π-Gate at the TMH3 of ND6 seen from the matrix side. Core subunits of the membrane arm are represented in shades of blue. The water chain in the central aqueous passage is interrupted by a ~9.1 Å gap. **c**, Density and fitted atomic model of the map region where C1-FDX (magenta) interacts with ND2 at the entrance to the potential aqueous half-channel that would connect the matrix to the central aqueous passage. The horizontal black line indicates that under our experimental conditions the channel is closed.

**Extended Data Fig. 7:**
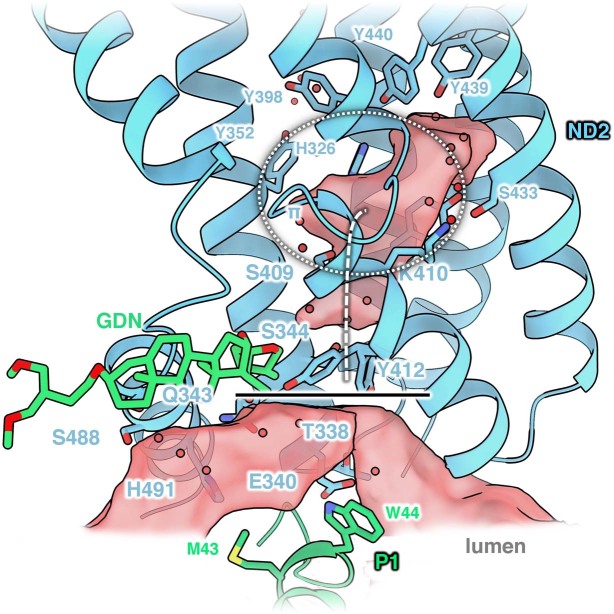
A potential lumenal half-channel at core subunit ND2. View along the membrane arm. In the *Arabidopsis* I + III_2_ supercomplex, a water-filled part of the central aqueous passage around the ND2 π-bulge (transparent red, white dotted ellipse) is blocked on the lumenal side (horizontal black line) by the sidechain of ND2 Y412 and an adjacent detergent molecule (GDN, green). Waters in red; GDN, glyco-diosgenin.

The redox reaction in the peripheral arm is energetically coupled to proton translocation in the membrane arm of complex I. Coupling is based on an aqueous passage that leads from the ubiquinone binding pocket to the ND5 subunit at the tip of the membrane arm. Ubiquinone reduction is thought to generate an electric impulse that is transferred along the aqueous passage and causes proton translocation through half-channels in the direction orthogonal to the membrane. The position, number and function of the half-channels in the membrane arm remain a matter of debate. The water structure in our high-resolution map of the *Arabidopsis* I + III_2_ supercomplex indicates three potential proton-entrance half-channels connecting the matrix to the aqueous passage in the membrane arm at core subunits ND2, ND4 and ND5 (Fig. 6a), in agreement with the structure of *Yarrowia* complex I (refs. 39, 40). The iron coordinated by C1-FDX is positioned close to the opening of the entrance half-channel at ND2 in a region that includes a number of ordered water molecules (Fig. 6c). The matrix channels at ND2 and ND4 seem to be closed under our experimental conditions, whereas the one of ND5 is open. On the lumenal side, two potential proton-exit half-channels from the central aqueous passage were identified. One is located at the tip of the membrane arm at ND5. This half-channel has also been seen in complex I from mammals and *Yarrowia lipolytica*^32,39,40^. In our structure, this channel is connected to the central aqueous passage while proton leakage from the open ND5 matrix half-channel is prevented by a 5–6 Å gap between ND5 H257 and T315. Our structure indicates a second lumenal half-channel at ND2 with a possible proton translocation pathway along a chain of water molecules and hydrophilic residues (Extended Data Fig. 7). This channel has been questioned. In *Yarrowia*, a weak water occupancy was suggested by molecular dynamics (MD) simulation at the same location^39^. A second half-channel on the lumenal side has been predicted on the basis of a *Yarrowia* mutant that lacks part of the membrane arm, including ND5, but still is partially active in proton translocation^41^. At the ND6 π-bulge, the hydrogen bond chain of the hydrophilic axis is interrupted (Fig. 6b). The π-bulge is identical in both our slightly different I + III_2_ supercomplex conformations, presumably due to the substrate-depleted conditions during sample preparation.

### A bound ubiquinone at the proton exit site of *Arabidopsis* complex III_2_

**Fig. 7:**
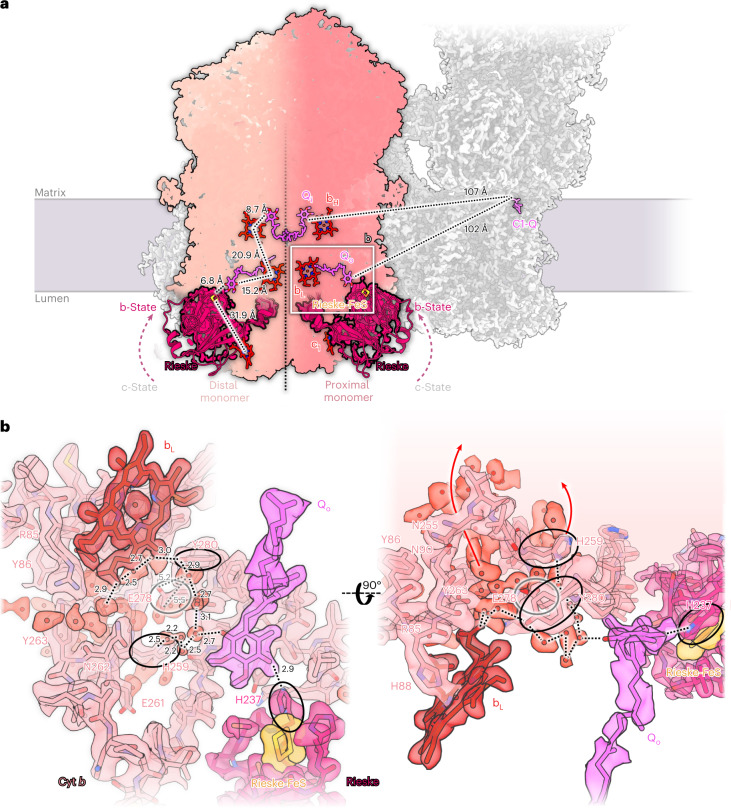
Mechanistic insights into *Arabidopsis* complex III_2_. **a**, Overview of complex III cofactors involved in respiratory electron transport. Each monomer binds haems c_1_, b_H_, b_L_ (red), a Rieske FeS cluster (orange) and a Q (magenta) at the reduction/oxidation site (Q_i_ and Q_o_). In both monomers of complex III_2_, the Rieske head domain (dark red) is found in the b state. Complex III monomers proximal or distal to the ubiquinone binding site of complex I (C1-Q) are shown in different shades of pink divided by a black dotted line. Distances between the cofactors are shown for the distal complex III monomer. Distances from Q_i_ and Q_o_ to the quinol binding site in complex I (C1-Q) are shown for the proximal monomer. **b**, Detailed view of the proximal Q_o_ site. Hydrogen network for release of the two protons during ubiquinol oxidation at the Q_o_ site are shown by black dotted lines. One proton is transferred along a chain of water molecules (light red) via Cyt *b* Y280 and H259; the other can be transferred directly to H237 of the Rieske head domain. The three participating sidechains are indicated by black ellipses. Cyt *b* E278 (grey) may be involved in proton translocation, faces away from the bound native Q and is not part of a proton pathway (grey dotted lines). Red arrows indicate routes for proton release to the bulk solvent of the cristae lumen. For further details, see Extended Data Figs. 8 and 9.

Each monomer of complex III_2_ has two binding sites for Q known as the Q-oxidation (Q_o_) and the Q-reduction site Q_i_^42^. In the structure of the I + III_2_ ovine supercomplex, Q densities were detected at only three of the four Q binding sites^25^. This was interpreted as a symmetry break in complex III_2_ and suggested to be relevant for supercomplex function. However, the *Arabidopsis* I + III_2_ supercomplex has Q molecules bound in all four sites, all at distances of 100–150 Å to the Q binding site of complex I (Fig. 7a).

**Extended Data Fig. 8:**
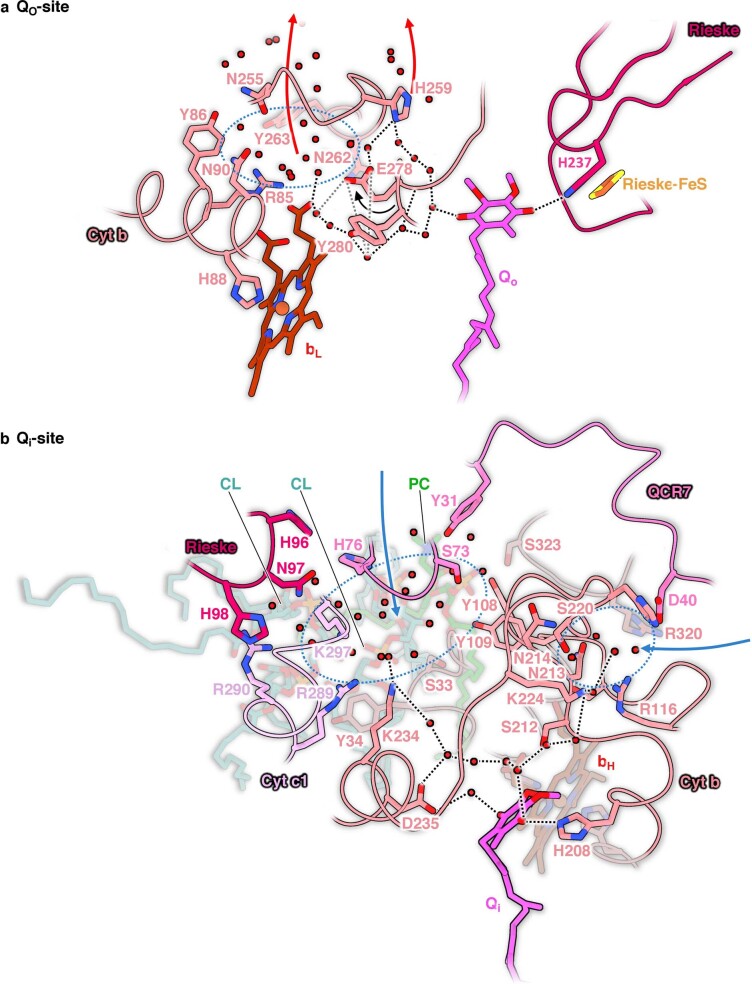
Architecture of the ubiquinol/ubiquinone (Q) binding sites in *Arabidopsis* complex III_2_. **a**, The Q_o_ site. One proton from bound ubiquinol can be transferred directly to H237 of the Rieske head domain in the b-state (black dotted line). The second proton can be released into the bulk solvent of the lumen (red arrows) along a chain of water molecules towards Cyt *b* H259 or towards a pool of water molecules (dotted blue ellipse) involving Cyt *b* Y280 and the heme b_L_ propionate group. The sidechain of Cyt *b* E278 of the Q_o_ motif faces away from the ubiquinol (black arrow) and its carboxylate group is not in hydrogen bond distance to the substrate or the water chain (grey dotted lines). **b**, The Q_i_ site. Proton transfer from the bulk solvent of the matrix (blue arrows) towards a carbonyl group of bound ubiquinon can proceed via two hydrogen bond networks from clusters of water molecules (blue ellipses). One of them is surrounded by subunit QCR7, the N-terminal part of the Rieske protein, subunit Cyt *c*_*1*_, Cyt *b* and three bound lipids (two cardiolipins, CL; one phosphatidylcholine, PC). From this pool, protons can be accepted by Cyt *b* K234 from where they can pass to the bound ubiquinone across Cyt *b* D235. The other proton transfer pathway involves Cyt *b* K224. Our structure indicates that Cyt *b* H208 participates in proton transfer by forming a direct hydrogen bond to ubiquinone. Red dots, water molecules; black dotted lines, hydrogen bonds. Colors of the protein subunits (Cyt c_1_, Cyt b, Rieske, QCR7) as in Fig. 2. Q is drawn in pink.

**Extended Data Fig. 9:**
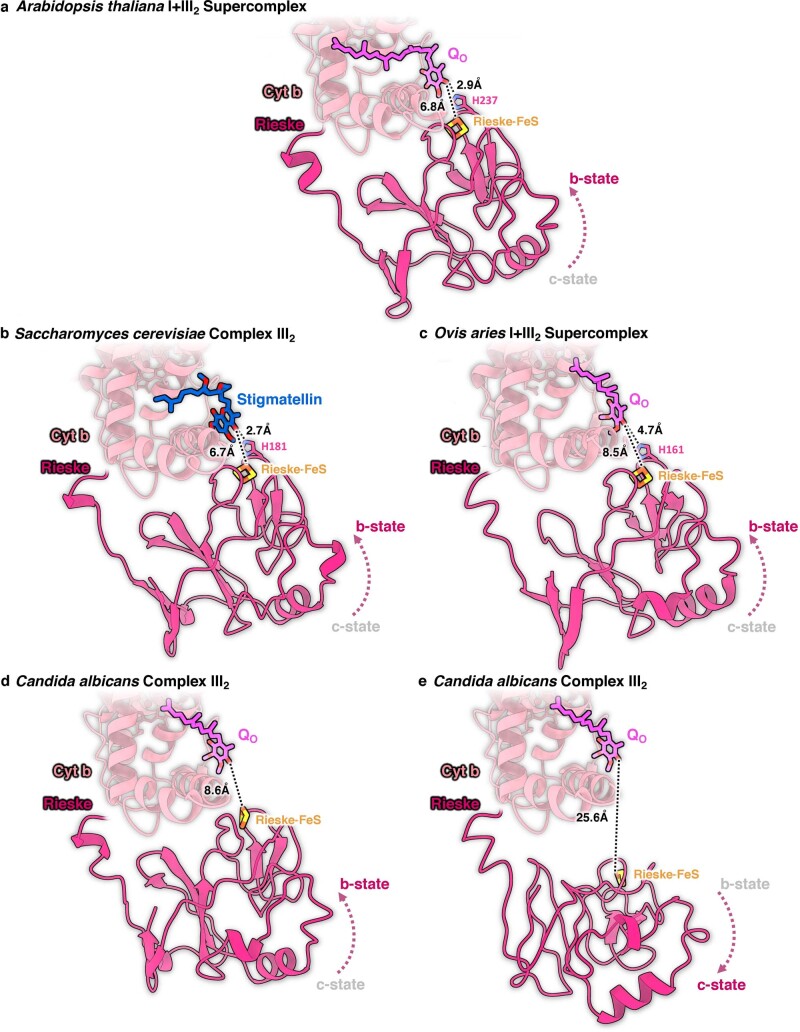
Positions of the Rieske head domain in complex III structures. In the *Arabidopis* I + III_2_ supercomplex **(a)**, the Rieske head domain (magenta) is locked in the b-state with its FeS cluster positioned 6.8 Å and H237 2.9 Å away from the bound ubiquinone/ubiquinol that accepts the electron. These distances resemble those in the stigmatellin-bound complex III_2_ crystal structure of *Saccharomyces cerevisiae* **(b)** and are smaller than in the b-state in the cryo-EM structures of the *Ovis aries* (sheep) I + III_2_ supercomplex **(c)** and of the *Candida albicans* complex III_2_ **(d)** where the Rieske head group was also resolved in the c-state **(e)**. Ubiquinone/ubiquinol is shown in pink, the inhibitor Stigmatellin in blue; distances between Q_o_/Rieske FeS group and Q_o_/FeS coordinating histidine are indicated by black dotted lines. PDB accession codes: *Saccharomyces*, 1EZV^43^; *Ovis*, 6Q9E^25^; *Candida* b-state, 7RJB, and *Candida* c-state, 7RJD^81^. Sidechains of the Rieske head domain histidine are not present in the *Candida* models.

The exact details of proton release and electron transfer at Q_o_ have long been a matter of debate, because there was no high-resolution structure of complex III with a bound Q in this site. Our 2 Å structure now shows the bound, co-purified Q substrate in each of the two Q_o_ sites of the complex III dimer and surrounding water molecules in close-to-atomic detail. Both Rieske head domains assume the b-state, with the Rieske FeS cluster positioned 6.8 Å away from the bound Q_o_, and the FeS-coordinating H237 of the Rieske domain interacting with the Q_o_ head group via a 2.9 Å hydrogen bond (Fig. 7a and Extended Data Figs. 8 and 9). These distances are in excellent agreement with the structures of yeast complex III_2_ with bound inhibitors stigmatellin or HHDBT^43,44^. In this distal binding position relative to haem b_L_, electrons can easily pass from Q_o_ to the Rieske iron–sulphur cluster in the course of the Q cycle^45^. During the cycle, protons from the quinol are released to the cristae lumen. In our structure, as in the stigmatellin- and HHDBT-bound yeast complex, one proton can be directly transferred to H237 of the Rieske head domain. The second proton can escape via a short chain of water molecules to Cyt *b* H259 and then to the cristae lumen. An alternative, longer path would lead via Cyt *b* Y280 and the b_L_ propionate group to a water cluster surrounded by conserved polar residues of Cyt *b* (R85, Y86, N90, N255, Y263 and N262). Either pathway would enable proton release to the bulk solvent without involving Cyt *b* E278 in the Q_o_ motif. This residue was suggested to play an important role in proton release because in the yeast complex it interacts directly with stigmatellin. However, in the *Arabidopsis* supercomplex, E278 does not interact with Q_o_ and only contributes to a water chain via its backbone nitrogen atom. This concurs with the HHDBT-inhibited yeast complex, where the inhibitor is thought to resemble the deprotonated ubisemiquinone anion state of Q. On the basis of our Q_o_ site structure, we propose that one proton is released via H259 in subunit Cyt *b* and the other is taken up by H237 in the Rieske domain.

### The MPP module of complex III_2_

In plants, complex III_2_ contains an active MPP domain protruding into the mitochondrial matrix. Complex III_2_ thus has a dual function in respiratory electron transport and in the maturation of proteins imported into mitochondria^46^. In our structure of the *Arabidopsis* respiratory I + III_2_ supercomplex, subunit MPP-β of each complex III monomer contains a bound Zn ion as a cofactor for peptidase activity (Fig. 2). The Zn is coordinated by two histidines (H141 and H145) and a glutamate (E221). In addition, the map indicates a potentially coordinating water molecule (Extended Data Fig. 2). This water molecule is known to be catalytically relevant as it performs a nucleophilic attack on the carbonyl carbon of the substrate peptide bond^47^. However, the adjacent glycine-rich loop of subunit MPP-α is not well resolved. This loop is flexible and involved in substrate binding and product release^48^. The weak cryo-EM density for this region would thus indicate an active MPP domain. Due to the distance between the MPP active site and the sites involved in respiratory electron transport, it has been assumed that the two complex III_2_ functions are independent^49^. On the basis of the recently identified structure of mung bean complex III_2_, a potential interplay between respiratory and peptide processing functions of plant complex III_2_ has been conjectured, because of a perceived long-range elongation or contraction motion within or between the two monomers^20^. In contrast, complex III_2_ in the *Arabidopsis* supercomplex does not appear to be intrinsically flexible, but it may adopt different orientations relative to complex I (Extended Data Figs. 3 and 4).

## Discussion

The 2 Å cryo-EM structure of the *Arabidopsis* respiratory I + III_2_ supercomplex provides detailed insights into how complexes I and III_2_ associate in the mitochondrial inner membrane (Figs. 1–4). Supercomplex formation involves protein–protein interactions at three distinct sites (Fig. 4), which explains the unusual stability of the plant supercomplex^21^. In *Arabidopsis* complex I, a defined conformation of the membrane arm promotes supercomplex formation. This conformation requires B14.7 and C1-FDX, which both interact with the C-terminal loop of ND5, thereby increasing the curvature of complex I in the membrane plane (Fig. 3). The importance of B14.7 and C1-FDX for supercomplex formation in *Arabidopsis* has recently been deduced from genetic experiments^50^. In mammalian and fungal complex I, B14.7 is likewise required for membrane arm stability^51,52^. In addition, the newly defined plant-specific subunit P9 contributes to the tight interaction of complexes I and III_2_ in *Arabidopsis*.

The coupling of NADH:ubiquinone oxidoreduction in the peripheral arm of complex I with proton translocation in the membrane arm may depend on long-range conformational changes^32,39^. Two conformations were described for the free form of *Arabidopsis* complex I (ref. 15), which differ in the angle between the peripheral and membrane arms. An open conformation (angle between the arms 112°) and a closed conformation (angle 106°) were reported. The C1-FDX subunit is not well defined or absent in the open conformation. On the basis of these observations, a role of C1-FDX in regulating complex I activity by adjusting the angle between the two complex I arms has been suggested^15^. However, in the *Arabidopsis* supercomplex, these two conformations are not evident. The two conformations of the supercomplex-bound form of complex I we describe in our present study are very similar and both correspond to the closed state with respect to the inter-arm angle. This angle ranges from 106° to 108° in the two conformations, and the C1-FDX subunit is present and well defined in both (Extended Data Fig. 3). In the *Arabidopsis* supercomplex, we do not find the open inter-arm conformation of complex I. Recently, the relevance of the inter-arm angle for complex I function has been questioned^53^. Rather than assigning a functional role to C1-FDX, we now conclude that the C1-FDX stabilizes complex I and that the open conformation of plant complex I (ref. 15) might be a destabilized form or assembly intermediate. However, on the basis of the supercomplex structure it seems likely that C1-FDX has an effect on proton translocation through the membrane arm, as the C1-FDX iron is located diagonally next to the proton-entry half-channel of ND2 on the matrix side of the membrane (Fig. 6a,c). It should be noted that protons are released close to this site by the activity of the carbonic anhdrase domain. Moreover, C1-FDX contributes to γCA-CoA-X binding in the γCA domain (Fig. 5a and Extended Data Fig. 5). Further investigations of C1-FDX, for example, by site-directed mutagenesis, will be necessary to clarify its role in complex I and the supercomplex.

We obtained new insights into the heterotrimeric γCA domain of complex I (Fig. 5), which is not present in mammals or fungi. The catalytic site at the γCA1–γCA2 interface perfectly resembles the active site of γCA of the bacterial CamH subclass (Extended Data Fig. 6). The two other catalytic sites lack one or two histidines for zinc binding. We now discovered a crotonyl- or butyryl-CoA at the γCA2–γCAL2 interface (Fig. 5a and Extended Data Fig. 5). Crotonyl-CoA might coordinate a CO_2_ molecule at this otherwise incomplete catalytic site. It is known that crotonyl-CoA can be carboxylated by crotonyl-CoA carboxylase/reductase using NADPH as a cofactor^35^. Further investigations, for example by site-directed mutagenesis, are needed to establish which chemical reaction is catalysed at the γCA2–γCAL2 interface of the γCA domain.

The physiological role of respiratory supercomplexes is still a matter of debate^54–56^. Direct channelling of ubiquinol between the Q-reduction site of complex I and the Q-oxidation site of complex III_2_ can be excluded, as the two substrate-binding sites are not in close proximity. Nevertheless, ubiquinol transfer from complex I to complex III_2_ might be facilitated by shorter diffusion distances between the substrate binding sites of the two complexes within the supercomplex. Also, supercomplex formation appears to promote respiratory chain function at other levels, for example, through the stability of individual complexes, reduction of ROS production or prevention of non-specific protein aggregation within the inner mitochondrial membrane. The I + III_2_ supercomplex of *Arabidopsis* clearly stabilizes the two component complexes.

### Concluding statement

This work is published together with a study on the cryo-EM structure of supercomplex I + III_2_ from *Vigna radiata*^57^. No experimental data or manuscript versions were exchanged between the groups before the papers were accepted, such that the independent studies would better complement and validate one another.

## Methods

### Plant material

*Arabidopsis thaliana* was cultivated as described^15^. A cell suspension culture was prepared from green leaves as previously described^15^.

### Isolation of mitochondria and purification of the I + III_2_ supercomplex

Mitochondria were isolated from *A. thaliana* cells as described^15^. Freshly prepared mitochondrial pellets (~100 mg, corresponding to ~10 mg mitochondrial protein) were suspended in 10 ml digitonin solubilization buffer (30 mM HEPES, pH 7.4, 150 mM potassium acetate and 5% (w/v) digitonin) and incubated for 15 min on ice. After centrifugation for 10 min at 4 °C, solubilized protein complexes were transferred onto sucrose gradients (0.3 M to 1.5 M sucrose in gradient buffer (30 mM HEPES, pH 7.8, 150 mM potassium acetate and 0.1% (w/v) digitonin)) and separated by ultracentrifugation at 146,000*g* and 4 °C for 20 h. Subsequently, sucrose gradients were fractionated and protein contents of relevant fractions were monitored by one-dimensional (1D) blue-native polyacrylamide gel electrophoresis^58^. The I + III_2_ supercomplex accumulates at a sucrose concentration of ~1 M (Supplementary Fig. 1a). The average protein concentration of the fractions was ~0.5 µg µl^−1^.

The I + III_2_ supercomplex was further purified by size-exclusion chromatography (Supplementary Fig. 1b). At this step, Foxglove digitonin (Merck) was replaced by synthetic digitonin (GDN, Anatrace). Supercomplex containing fractions from sucrose density ultracentrifugation were pooled and incubated for 30 min at 4 °C in cryo-EM buffer (30 mM Tris–HCl pH 7.4, 60 mM NaCl and 0,02% (w/v) GDN). They were then diluted 1:200 in the same buffer during concentration to a final volume of 50 µl using an Amicon Ultra 15 centrifugal filter unit (Merck) and a Vivaspin 500 concentrator (Merck) with 100 kDa molecular weight cut-off. The concentrated sample was loaded onto a Superose 6 Increase 3.2/300 size-exclusion chromatography column (Cytiva). Fractions containing supercomplex were eluted in cryo-EM buffer and directly used for EM grid preparation (see below).

### Oxidoreductase activity of isolated I + III_2_ supercomplex from *A. thaliana*

*Arabidopsis* I + III_2_ supercomplex purified by sucrose gradient ultracentrifugation was tested for activity, using an established protocol^59^. The assay was carried out in 25 mM potassium phosphate (pH 7.2), 5 mM magnesium chloride, 260 µM NADH, 67 µM decylubiquinone (Sigma-Aldrich), 0.1 mM horse cytochrome *c* (Sigma-Aldrich) and 3,000 U ml^−1^ superoxide dismutase to ensure that cytochrome *c* remained oxidized^25^. The assay volume was 170 µl. Four different conditions with or without cytochrome *c* and decylubiquinone were tested (Supplementary Fig. 5). For each condition, measurements of two blanks (0 µg I + III_2_ supercomplex) as well as two measurements each of 2 µg and 4 µg I + III_2_ supercomplex were performed. Activity measurements were carried out with a plate reader (Multiscan Sky, Thermo Fisher Scientific) at 340 nm (NADH absorbance; extinction coefficient of NADH: 6.22 mM^−1^ cm^−1^).

### Analysis of the I + III_2_ supercomplex by MS

Supercomplex-containing fractions from sucrose density ultracentrifugation were prepared for MS analysis via the single-pot-solid-phase-enhanced sample preparation (SP3) protocol^60^. Here we used a modified protocol^61^ adapted for the amount of available sample material. The sucrose gradient fractions were diluted in equal volumes of 2× SDT buffer (8% (w/v) sodium dodecyl sulphate, 0.2 M dithiothreitol and 0.2% Tris–HCl, pH 7.6) and incubated on a thermoshaker (TS-100, Kisker Biotech) for 1 h at 60 °C and 1,000 rpm. After centrifugation for 10 min at 20,000*g*, the supernatant was transferred into a new reaction tube and sonicated in a water bath for 10 min (Elmasonic S30, Elma). Proteins were alkylated via incubation in 20 mM iodoacetamide for 30 min at 600 rpm at room temperature in the dark. Alkylation was stopped by addition of 5 mM dithiothreitol.

Sera-Mag carboxylate-modified beads hydrophilic solids (GE Life Sciences) were combined 1:1 with hydrophobic solids (GE Life Sciences), and a total amount of 600 µg beads was added to each sample. Proteins were precipitated by addition of 70 µl ethanol (100%) and subsequent incubation for 10 min at 1,000 rpm at 24 °C. Beads were pelleted on a magnetic rack for 2 min, and proteins were washed three times with 140 µl of fresh 80% ethanol. After protein clean-up, beads were transferred in 80% ethanol into low-protein-binding tubes (Low Binding Micro Tubes, Sarstedt) and ethanol was removed on the magnetic racks.

Proteins were digested with 2 µg of sequencing-grade modified trypsin (V5111, Promega) in 50 mM ammonium bicarbonate at 37 °C at 1,000 rpm overnight in a total reaction volume of 60 µl. Trypsin activity was stopped the next day by addition of 1% (v/v) formic acid (FA). The pH of each sample was controlled and adjusted to <3.

Tryptic peptides were further cleaned via solid-phase extraction on SepPak Vac 1cc (50 mg) tC18 cartridges (Waters). Cartridges were wetted with 1 ml 100% acetonitrile and 1 ml 0.1% (v/v) FA in 50% (v/v) acetonitrile. Cartridge equilibration was performed by adding 2× 1 ml of 0.1% FA (v/v) in H_2_O. Acidified peptides (pH <3) were loaded onto the cartridges and washed two times with 0.1% FA (v/v) in H_2_O and eluted two times in 200 µl of 0.1% FA (v/v) in 50% (v/v) acetonitrile. Cleaned peptides were dried in a vacuum centrifuge and stored at −20 °C. Final peptide concentration was determined with the Pierce peptide quantification kit (Thermo Scientific) following the manufacturer’s instructions.

A nanoElute HPLC (Bruker Daltonics) was coupled to a timsTOF Pro ion-mobility spectrometry quadrupole time-of-flight mass spectrometer (Bruker). Peptides were reconstituted in 0.1% FA, and 400 ng peptides per sample were directly transferred onto an ‘Aurora’ reversed-phase analytical column with integrated emitter tip (25 cm × 75 μm inner diameter, IonOpticks, Fitzroy). Peptides were separated on the analytical column at 50 °C via a 70 min gradient (solvent A: 0.1% FA; solvent B: 0.1% FA in 100% acetonitrile) at a flow rate of 300 nl min^−1^. A linear gradient from 2% to 37% B for the first 60 min was followed by a 10 min washing step at 95% B.

The timsTOF Pro mass spectrometer was operated in DDA PASEF mode, and the pre-installed method ‘DDA PASEF-standard_1.1sec_cycletime’ was used. Automatic recalibration of ion mobility before each sample run was activated. MS and MS/MS scan range was 100–1,700 *m*/*z*, the ion mobility range (1/K_0_) was 0.6–1.6 V s^−1^ cm^−2^. A polygon filtering was applied in the *m*/*z* and ion mobility area to exclude the low *m*/*z* of singly charged ions for PASEF precursor selection. Ramp and accumulation time was set to 100 ms to achieve close to 100% duty cycle. The number of PASEF ramps was set to 10 with a charge maximum of 5. The quadrupole isolation width was set to 2 for *m*/*z* = 700 and 3 for *m*/*z* = 800. Collision energy was 20 eV for ion mobility (1/K_0_) 0.6 V s^−1^ cm^−2^ and 59 eV for ion mobility (1/K_0_) 1.6 V s^−1^ cm^−2^, respectively.

### Evaluation of MS data

MaxQuant 2.0.3.0 (ref. 62) was used to query acquired MS/MS spectra against a modified TAIR10 database including models of mitochondrial and plastid genes after RNA editing to improve sequence coverage of affected proteins (see ref. 63). Default parameters were used with the following exception: calculation of iBAQ values^64^ was activated, the options ‘Log fit’ and ‘charge normalization’ were enabled. Identification transfer between individual runs via the ‘Match between runs’ feature was applied with the default parameters.

### Cryo-EM grid preparation

Quantifoil R 1.2/1.3 400 Cu grids were washed for 1 h in chloroform. They were coated with a 2 nm carbon layer using a Leica EM ACE600 high vacuum sputter coater or a graphene trivial transfer single layer (ACS Material LLC). Graphene-coated grids were pre-treated as described previously^65^ and functionalized with 20 µM 1-polybutyric acid or just glow discharged for 15 s at 15 mA. Carbon-coated grids were just glow discharged in the same way. Supercomplex was applied at a final concentration of 0.18 mg ml^−1^ and the grids were plunge-frozen in liquid ethane after blotting for 4.5 s with force 20 using a Mark Vitrobot IV operating at 10 °C and 70 % humidity. Grids were stored in liquid nitrogen.

### Data acquisition and image processing

Electron micrographs were collected at 300 kV with a Titan Krios G4i (Thermo Scientific) equipped with a cold field emission gun and a Falcon 4 detector operating in electron counting mode. The nominal magnification was 215,000×, corresponding to a pixel size of 0.573 Å. Electron-event representation (EER) movies consisting of 1,118 raw frames were recorded automatically with EPU software at an exposure rate of 3.4 e^−^ pixel^−1^ s^−1^ and a total dose of 50 e^−^ Å^−2^. Four datasets were collected, three from graphene grids and one from a carbon-coated grid. Data were processed separately. Movies of the single datasets were motion-corrected using MotionCor2 (ref. 66) and contrast transfer function (CTF) was estimated with CTFFind4.1.13 (ref. 67). After picking with crYOLO^68^, the particles were imported into Relion3.1.3 (ref. 69). Particles were binned to a pixel size of 2.558 Å, and an initial 3D classification was performed to clean the dataset. Next, a second 3D classification with a mask around the complex III dimer was performed to separate the supercomplex from unassociated complex I. Supercomplex particles were 3D-refined and re-extracted at the original pixel size of 0.573 Å. Two rounds of CTF refinement and Bayesian polishing were performed for each dataset, before they were merged. The final 3D reconstruction of the merged dataset indicated a resolution of 2.55 Å for the whole supercomplex. For an additional global non-uniform refinement, the merged particles were also imported into cryoSPARC v4.0.2 (ref. 70) and combined into a map with an overall resolution of 2.36 Å. Further processing was continued in RELION. For particle subtraction, the peripheral and membrane arm of complex I and the complex III dimer within the supercomplex were masked. Particle subtraction was followed by multibody-refinement steps for each of the separate domains. This resulted in a final resolution of 2.03 Å and 2.04 Å for the N and Q module of the CI peripheral arm, 2.13 Å and 2.29 Å for the P_P_ and P_D_ module of the CI membrane arm, and 2.29 Å and 2.25 Å for the MPP modules and the membrane part of complex III. Resolution estimates are based on the gold-standard Fourier shell correlation of two independent half-maps at the 0.143 cut-off^71,72^. The B-factor sharpened cryo-EM densities were further modified with the phenix.resolve_cryo_em tool^73^ using the refined maps, the two half-maps, the estimated molecular mass and a mask. Density modification improved the map quality to calculated resolutions of 1.93 Å, 1.89 Å, 2.02 Å and 2.14 Å for the N, Q, P_P_ and P_D_ module of complex I, and to 2.17 Å and 2.14 Å for the MPP module and membrane part of complex III. To separate different conformations of the *Arabidopsis* I + III_2_ supercomplex, focused 3D classification of the final consensus refinement was performed. Particles were first aligned by the complex I peripheral arm, using a local mask for initial 3D refinement. The particles were then 3D-classified by applying a soft mask around the membrane arm and complex III_2_ performing no further particle alignment with a value of *T* = 25. The two most dissimilar classes were further refined with a global mask, resulting in an overall resolution of 2.73 Å for conformation 1 and 2.80 Å for conformation 2. Final focused 3D refinement around the complex I peripheral arm, complex I membrane arm and complex III_2_ for each of the two conformations improved the resolution to 2.34 Å and 2.41 Å, respectively.

### Model building

The initial model for the *Arabidopsis* supercomplex was built using the complete model of the *A. thaliana* complex I (pdb: 7ARB) and homology models for each individual subunit of complex III that were created by the SWISS-MODEL server^74^. Individual subunits were then assembled using the complex III_2_ structure of *V. radiata* (pdb: 7JRG) as a template. Together, the complex I and assembled complex III_2_ model of *A. thaliana* were rigid-body fitted into the cryo-EM map of the supercomplex using UCSF Chimera^75^. Manual building was performed in Coot^76^ using the post-processed maps from Relion and the density-modified maps from Phenix with additional refinement with phenix.real_space_refine^77^. Water molecules were built automatically into the density-modified maps using the Segger Chimera SWIM tool^78^, and fits were checked manually. Model quality statistics were taken from phenix.validation_cryoEM and are summarized in Supplementary Table 3. Aqueous cavities were calculated with a 1.4 Å interior and a 3.5 Å exterior probe radius using Hollow^79^.

### Reporting summary

Further information on research design is available in the Nature Portfolio Reporting Summary linked to this article.

## Supplementary information

Supplementary Information Supplementary Figs. 1–6 and Tables 1–9.

Reporting Summary

Supplementary Video 1 Three-dimensional cryo-EM density map of the *Arabidopsis* I + III_2_ supercomplex at around 2 Å resolution with corresponding atomic model.

Supplementary Video 2 High-resolution map density around the N2 iron–sulphur cluster in the peripheral arm of complex I.

## Data Availability

The MS proteomics data of the mitochondrial I + III_2_ supercomplex from *Arabidopsis thaliana* are available at the PRIDE repository (https://www.ebi.ac.uk/pride/), dataset identifier PXD036482. Cryo-EM density maps and atomic models of the I + III_2_ supercomplex from *Arabidopsis thaliana* are available at the EM Data Bank (EMDB, https://www.ebi.ac.uk/emdb/, accessions EMD-15998, EMD-15999, EMD-16000, EMD-16003, EMD-16007, EMD-16008, EMD-16168, EMD-16171, EMD-16172) and Protein Data Bank (PDB, https://www.rcsb.org/, accessions 8BED, 8BEE, 8BEF, 8BEH, 8BEL, 8BEP, 8BPX, 8BQ5 and 8BQ6). Details are given in Supplementary Tables 2 and 3.
