# Supplementary material for: Cryo-EM structure of the respiratory I + III2 supercomplex from Arabidopsis thaliana at 2 Å resolution
Source: Nat Plants. 2022 Dec 30;9(1):142–56. doi: 10.1038/s41477-022-01308-6 (PMC9873573; doi:10.1038/s41477-022-01308-6)
Supplement: Supplementary file 1 — Supplementary Figs. 1–6 and Tables 1–9. [file 41477_2022_1308_MOESM1_ESM.pdf]

nature plants

Article https://doi.org/10.1038/s41477-022-01308-6

Cryo-EM structure of the respiratory I + III_2 supercomplex from *Arabidopsis thaliana* at 2 Å resolution

In the format provided by the authors and unedited

Supplementary Data

Klusch et al. (2022). Cryo-EM structure of the respiratory I + III_2 supercomplex from *Arabidopsis thaliana* at 2 Å resolution

Nature Plants

List of Supplementary Figures and Tables

Supplementary Figures

Supp. Fig. 1: Purification of the *Arabidopsis* I+III_2 supercomplex
Supp. Fig. 2: Cryo-EM data acquisition and image processing of the *Arabidopsis* I+III_2 supercomplex
Supp. Fig. 3: Fourier shell correlation (FSC) curves for the final post-processed and density-modified multibody maps
Supp. Fig. 4: Fourier shell correlation (FSC) curves for final post-processed conformation maps
Supp. Fig. 5: Activity of the purified I+III_2 supercomplex from *Arabidopsis*
Supp. Fig. 6: Comparison of the I+III_2 supercomplex from *Arabidopsis* obtained by single particle negative stain EM (Dudkina et al. 2005) and by cryo-EM (this study)

Supplementary Tables

Supp. Tab. 1: EM Statistics
Supp. Tab. 2: Cryo-EM map identifiers and quality statistics
Supp. Tab. 3: Model identifiers and quality statistics
Supp. Tab. 4: Subunits of the *Arabidopsis* I+III_2 supercomplex identified by mass spectrometry
Supp. Tab. 5: Nomenclature of complex I subunits in *Arabidopsis* and other model species
Supp. Tab. 6: Nomenclature of complex III subunits in *Arabidopsis* and other model species
Supp. Tab. 7: Calculated properties of the 48 complex I subunits of *Arabidopsis*
Supp. Tab. 8: Calculated properties of the 10 complex III subunits of *Arabidopsis*
Supp. Tab. 9: Amino acid composition of *Arabidopsis* complex III_2 (2x10 subunits) and *Arabidopsis* complex I (48 subunits)

Supplementary Videos (extra files)

Supp. Video 1: Three dimensional cryo-EM density map of the *Arabidopsis* I+III_2 supercomplex at around 2 Å resolution with corresponding atomic model
Supp. Video 2: High resolution map density around the N2 iron sulfur cluster in the peripheral arm of complex I.

References

Supplementary Figures

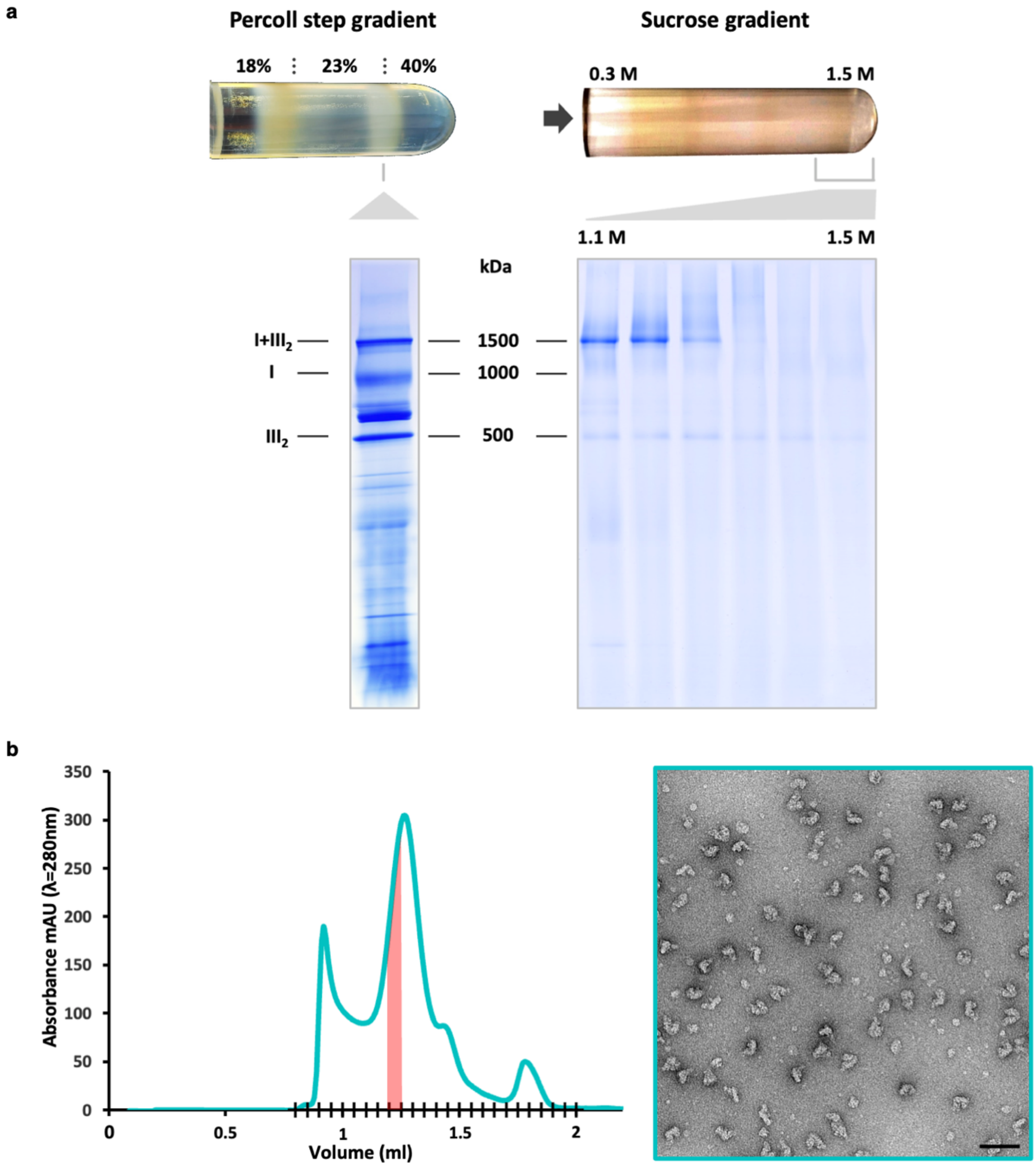

Supplementary Fig. 1: Purification of the *Arabidopsis* I+III$_2$ supercomplex.

a, *Arabidopsis* mitochondria were purified by Percoll step density gradient centrifugation (left). Mitochondria accumulated at the 23%/40% Percoll interface (for details see Materials and Methods). Subsequently, mitochondria were solubilized with digitonin and protein complexes were separated by sucrose gradient ultracentrifugation (right). The protein composition of gradient fractions was monitored by one-dimensional Blue native PAGE (gels below gradients). Fractions at 1.1 M sucrose were highly enriched in I+III$_2$ supercomplex. **b,** For further purification, digitonin was replaced by the synthetic digitonin analogue glyco-diosgenin (GDN), first by buffer exchange during step concentration, followed by size-exclusion chromatography. Negative-stain EM indicates a majority of intact I+III$_2$ supercomplex particles. Scale bar, 50 nm.

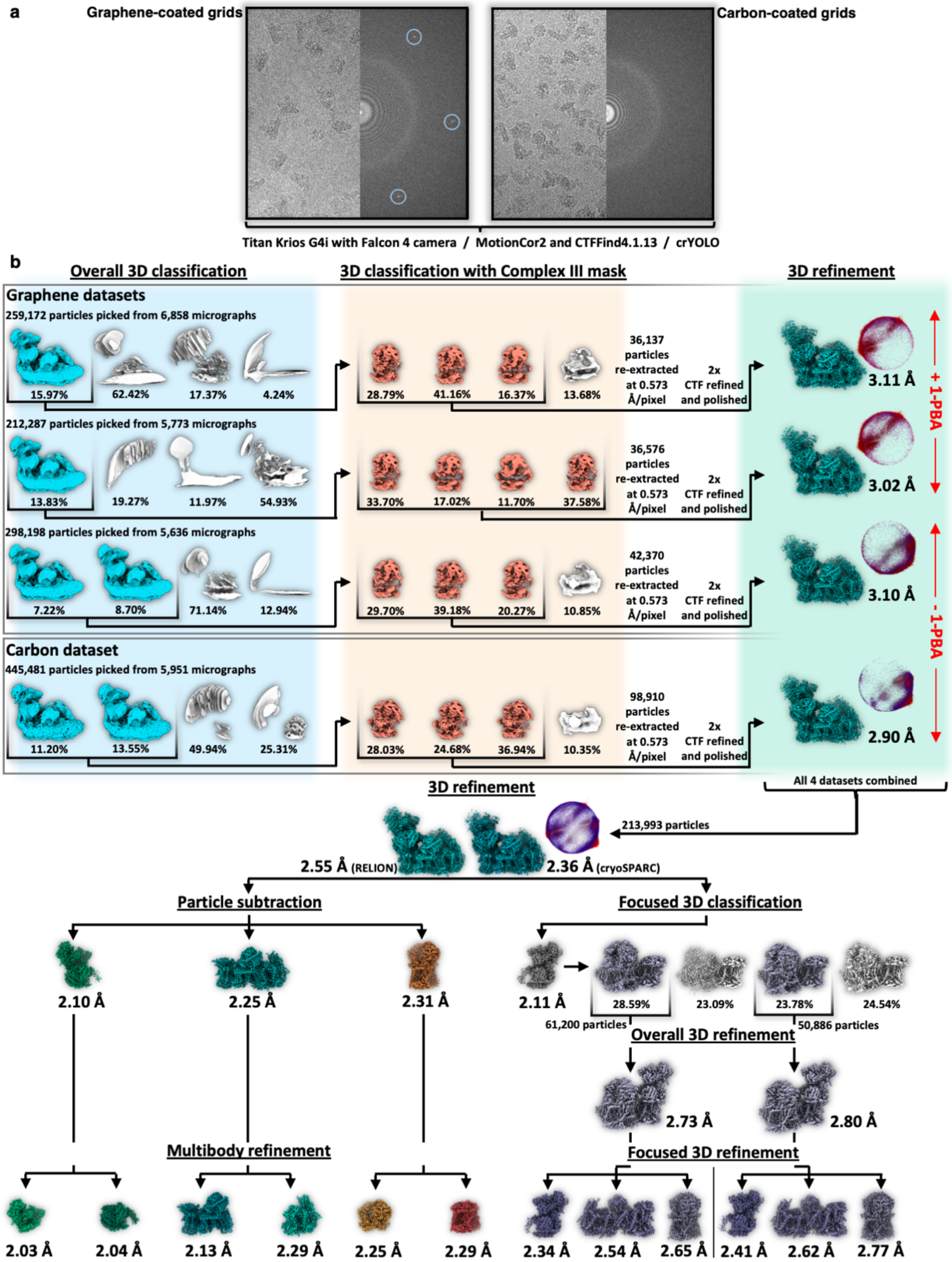

Supplementary Fig. 2: Cryo-EM data acquisition and image processing of the *Arabidopsis* I+III$_2$ supercomplex. a, Images were recorded with a Titan Krios G4i microscope equipped with a cold field emission gun and a Falcon 4 camera in electron-counting mode. Images were collected from graphene or carbon-coated grids. The reciprocal lattice of graphene in the Fourier transform is indicated by circles. **b,** Four datasets were collected, three on graphene, one on carbon coated grids. Support films were treated with (+ 1-PBA) or without (- 1-PBA) 1-polybutyric acid, which improved the angular distribution. After initial 3D classification, complex III$_2$ was masked to exclude unassociated complex I. The polished and CTF-refined datasets were combined, resulting in an overall resolution of 2.55 Å in RELION. The resolution was further improved by particle subtraction and multibody refinement, resulting in a resolution of 2.03 Å for the complex I peripheral arm, 2.13 Å for the complex I membrane arm, and up to 2.25 Å for the complex III dimer. To separate different conformations, particles were first aligned by the peripheral arm of complex I. Subsequently, focussed 3D classification was carried out with a mask around the complex I membrane arm and complex III$_2$. The two most different classes were refined further, resulting in two classes with overall resolutions of 2.73 Å and 2.80 Å. Focused 3D refinement around the complex I peripheral arm, complex I membrane arm and complex III$_2$, improved the resolution of the two conformations to 2.34 Å and 2.41 Å, respectively.

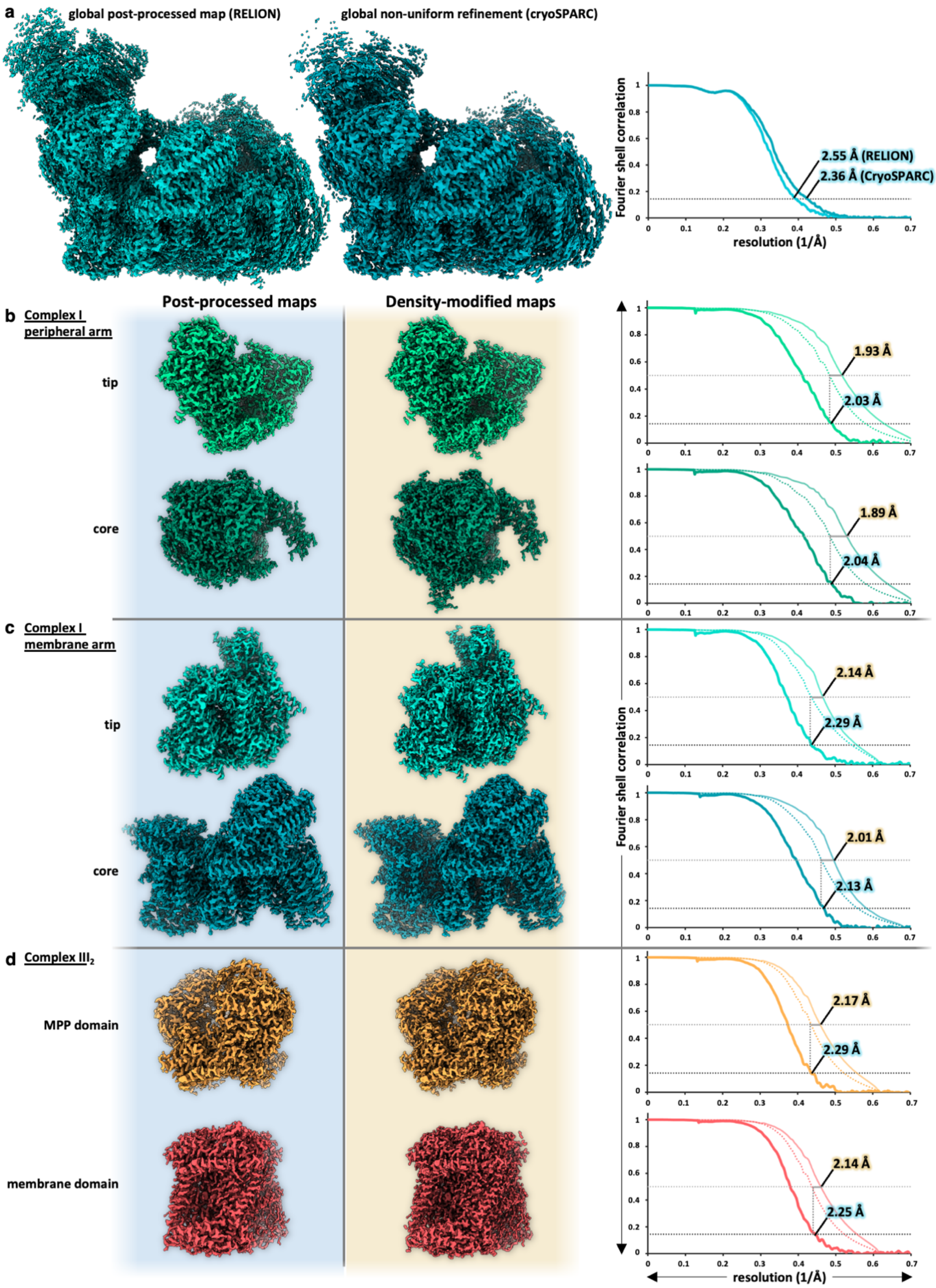

Supplementary Fig. 3: Fourier shell correlation (FSC) curves for final post-processed and density-modified multibody maps. a, Overall map and gold-standard FSC curve. **b-d**, Final maps of the complex I peripheral arm (**b**), complex I membrane arm (**c**) and complex III_2 (**d**). Gold-standard FSCs of post-processing steps to estimate map resolution by the 0.143 criterion are indicated by continuous curves. Density modification improved the map quality. The corresponding estimated resolution-dependent map accuracy values (FSC_{ref}) of the original and modified map are indicated by dashed or faint continuous curves. Straight grey lines indicate the resolution improvement at 0.5 FSC.

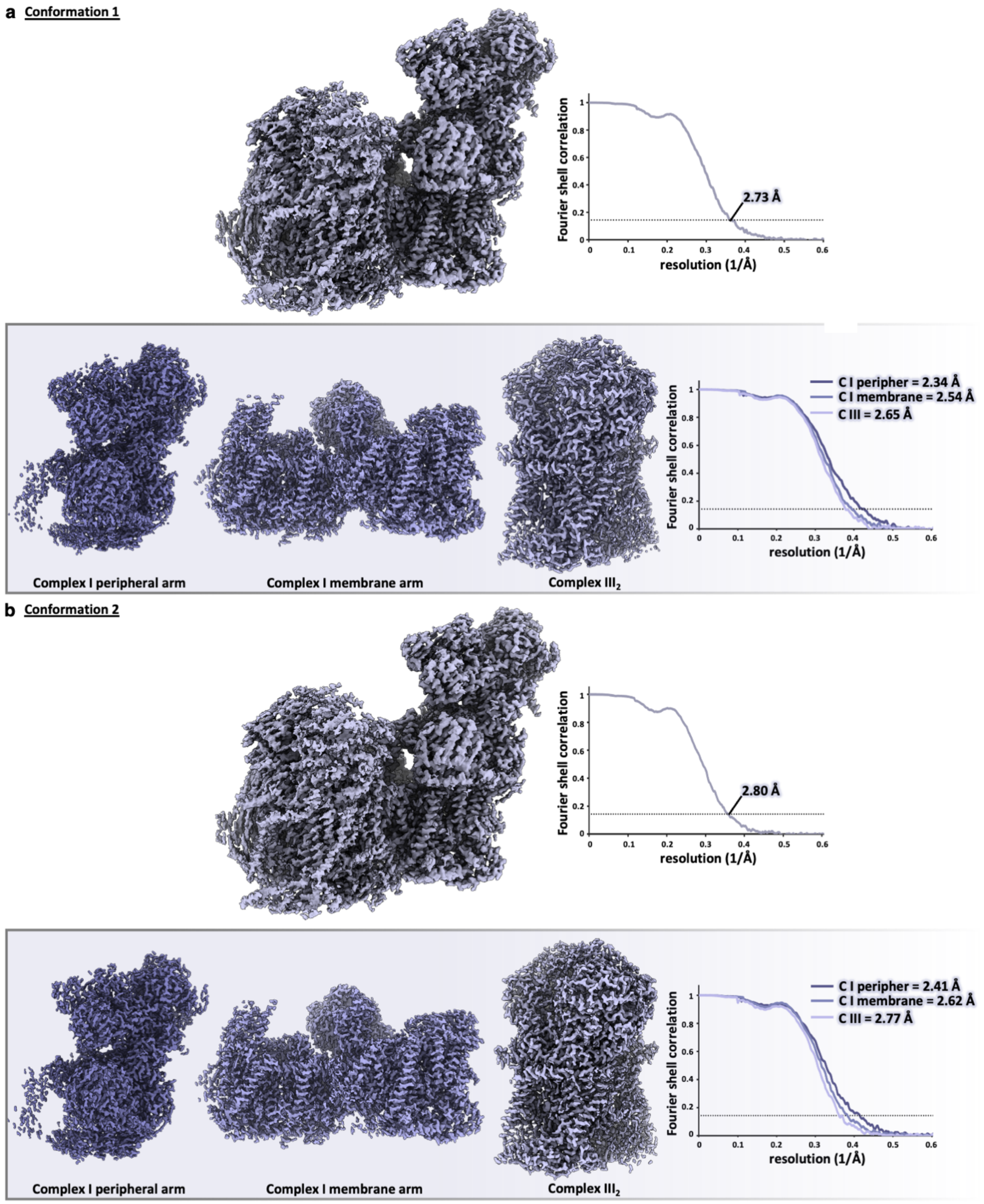

Supplementary Fig. 4: Fourier shell correlation (FSC) curves for final post-processed conformations. Overall map of the *Arabidopsis* I+III_2 supercomplex in conformation 1 (**a**) and conformation 2 (**b**) with corresponding focused 3D refined maps of the complex I peripheral arm, complex I membrane arm and dimeric complex III_2. Resolution of the final maps was estimated by gold-standard FSC curves with the 0.143 criterion.

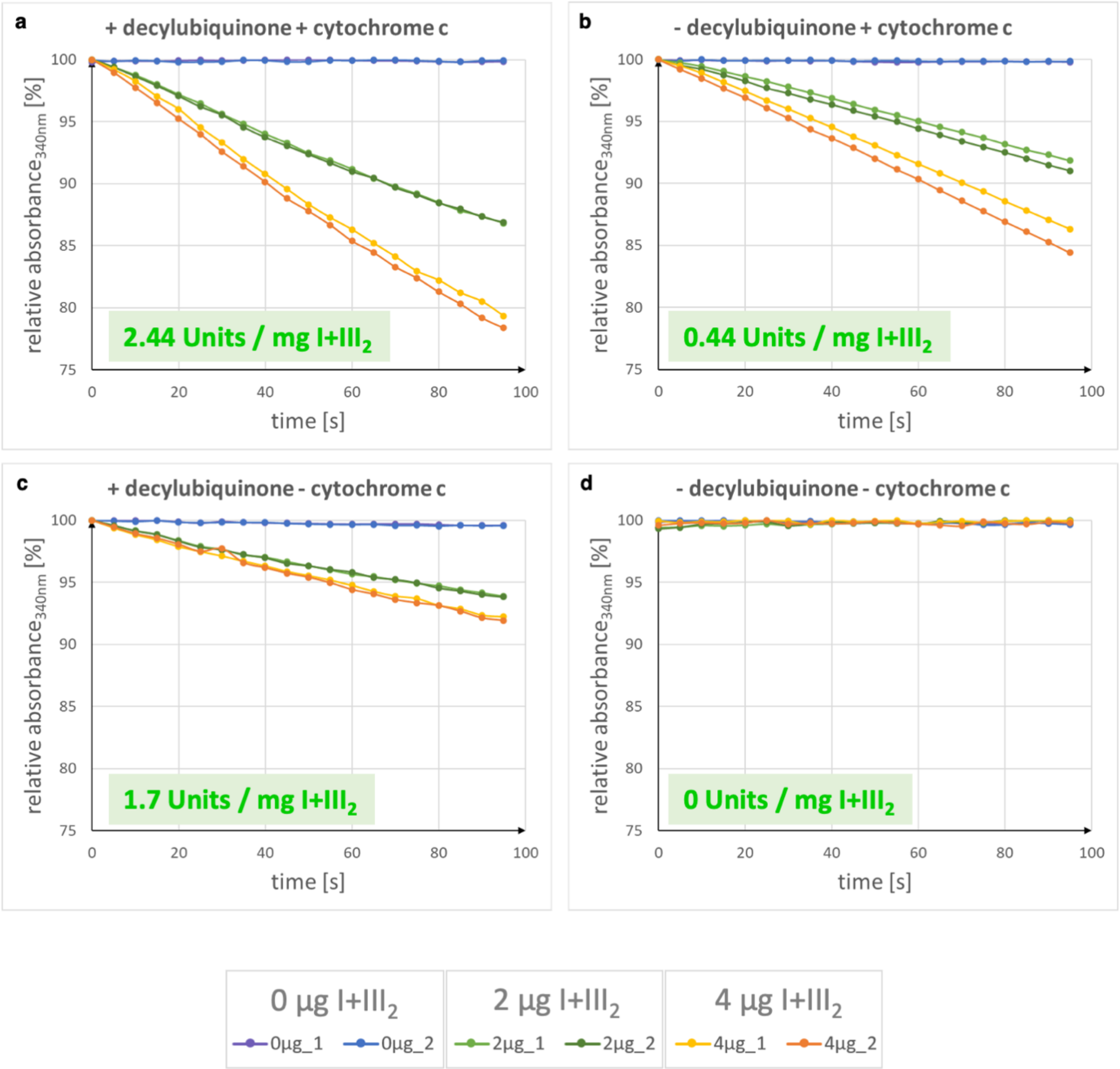

Supplementary Fig. 5: Activity of the purified I+III$_2$ supercomplex from *Arabidopsis*. Activity was measured (**a**) with decylubiquinone and cytochrome; (**b**) with decylubiquinone but without cytochrome *c*; (**c**) without decylubiquinone but with cytochrome *c*; and (**d**) without both decylubiquinone or cytochrome *c*. Absorbance was determined for >90 s at 340 nm (NADH). All four conditions were replicated twice. (i) A blank sample (0 µg I+III$_2$ supercomplex; 0µg_1, 0µg_2), (ii) 2 µg I+III$_2$ supercomplex (2µg_1, 2µg_2), (iii) 4 µg I+III$_2$ supercomplex (4µg_1, 4µg_2). Average enzyme activity (units / mg I+III$_2$ supercomplex) determined in each setup are given in green at the bottom of the diagrams. For experimental details see Materials and Methods.

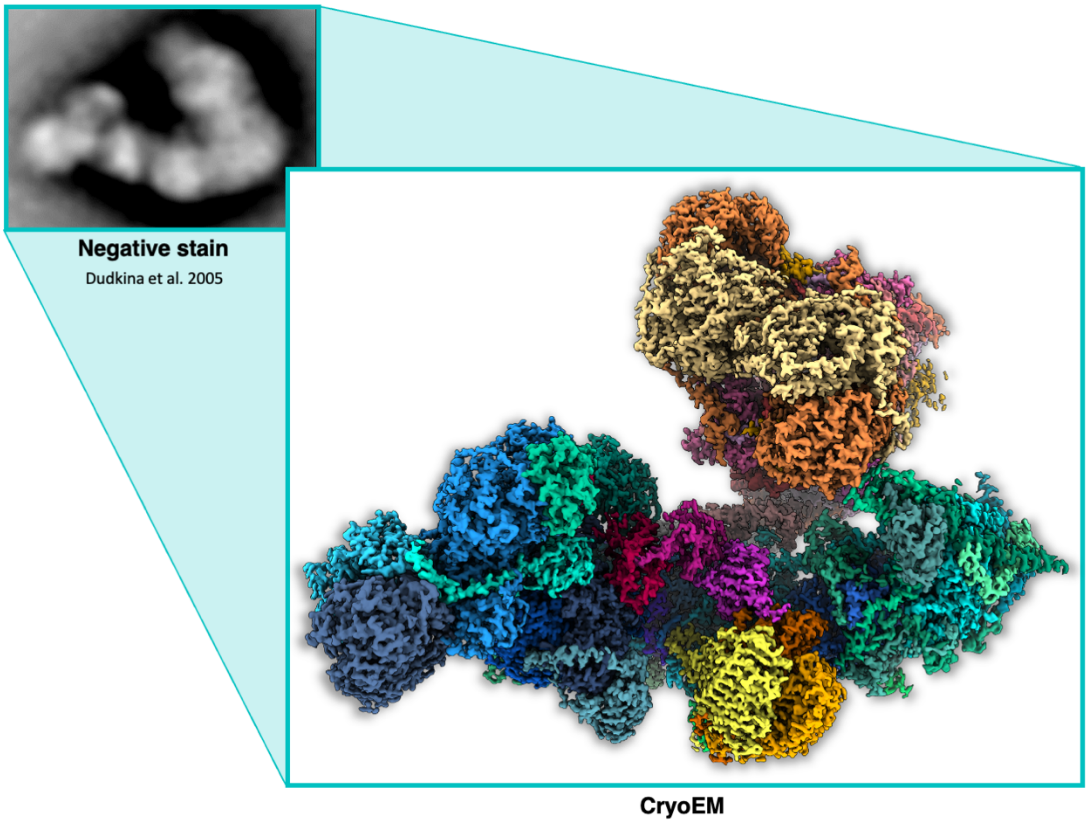

Supplementary Fig. 6: Comparison of the I+III$_2$ supercomplex from *Arabidopsis* obtained by negative stain EM[1] and by cryo-EM (this study).

Supplementary Tables

Supplementary Table 1: EM Statistics

**Data Collection**	
Electron Microscope	Titan Krios G4i
Camera	Falcon 4 (electron-counting mode)
Data collection software	EPU with Aberration-free image shift (AFIS)
Voltage	300 kV
Nominal magnification	215,000 x
Calibrated physical pixel size	0.573 Å
Total exposure	50 $e^{-}Å^{-2}$
Exposure rate	3.4 $e^{-}pixel^{-1}s^{-1}$
Number of frames	1,118 raw frames
Defocus range	−0.5 to −1.5 µm
**Image Processing**	
Motion correction software	MotionCor2
CTF estimation software	CTFFind4.1.13
Particle selection software	crYOLO
Micrographs used	24,218 movies in EER format
Particles selected	1,215,138
Classification and refinement software	Relion3.1.3
Particles contributing to final dataset	213,993 (115,083 on graphene / 98,910 on carbon)
Density modification software	Phenix (phenix.resolve_cryo_em)
**Model Building**	
Modeling software	Coot
Refinement software	Phenix (phenix.real_space_refine)

Supplementary Table 2: Cryo-EM map identifiers and quality statistics

Description	PDB ID	EMDB ID	Resolution (0.143)	Applied B-factor	# of particles	Symm.
CI Peripheral arm tip	8BED	EMD-15998	2.03Å	-22.6	213,993	C1
CI Peripheral arm core	8BEE	EMD-15999	2.04 Å	-22.3	213,993	C1
CI Membrane arm core	8BEF	EMD-16000	2.13 Å	-26.8	213,993	C1
CI Membrane arm tip	8BEH	EMD-16003	2.29 Å	-29.3	213,993	C1
CIII Membrane domain	8BEL	EMD-16007	2.25 Å	-28.7	213,993	C1
CIII MPP domain	8BEP	EMD-16008	2.29 Å	-29.8	213,993	C1
Composite map	8BPX	EMD-16168	-	-	213,993	C1
Composite Conformation 1	8BQ5	EMD-16171	-	-	213,993	C1
Composite Conformation 2	8BQ6	EMD-16172	-	-	213,993	C1

Supplementary Table 3: Model identifiers and quality statistics

Description	PDB ID	EMDB ID	Residues built	RMS Bonds Length/Angles		Ramachandran Outliers (%)	Ramachandran favoured (%)	Rotamer outliers (%)	Clashscore	EMRinger score
CI Peripheral arm tip	8BED	EMD-15998	1,607	0.006	0.765	0.00	97.17	0.37	7.63	6.04
CI Peripheral arm core	8BEE	EMD-15999	1,627	0.010	1.129	0.00	96.01	0.85	8.27	6.25
CI Membrane arm core	8BEF	EMD-16000	3,170	0.012	1.008	0.00	97.88	0.86	12.16	5.43
CI Membrane arm tip	8BEH	EMD-16003	1,589	0.010	1.354	0.00	97.76	1.55	12.51	4.58
CIII Membrane domain	8BEL	EMD-16007	2,054	0.010	1.133	0.00	97.04	0.98	11.71	4.63
CIII MPP domain	8BEP	EMD-16008	2,140	0.007	0.970	0.00	97.32	1.38	10.35	4.28
Composite map	8BPX	EMD-16168	12,187	0.010	1.157	0.01	97.23	1.01	13.03	5.01
Composite Conformation 1	8BQ5	EMD-16171	12,187	0.011	1.202	0.01	97.25	1.13	12.89	3.85
Composite Conformation 2	8BQ6	EMD-16172	12,187	0.010	1.209	0.01	97.24	0.97	12.57	3.74

I+III subunit	Accession	Unique peptides	Coverage [%]	Molecular mass	No. of residues	iBAQ ↓
MPP-β	AT3G02090.1	34	69.7	56.2	506	14856000
CA2	AT1G47260.1	15	57.2	29.9	277	11734000
MPP-α-1	AT1G51980.1	21	62.8	51.5	477	11376000
QCR7-1 (14 kDa)	AT4G32470.1	4	40.2	14.4	121	11152000
B16.6-2	AT2G33220.1	2	9.8	16.0	142	9665400
Cyt c1-2	AT3G27240.1	6	28	27.1	243	9368100
B14.5a	AT5G08060.1	7	72.5	14.9	130	9073200
B22	AT4G34700.1	10	72.6	13.5	116	7855500
ND9 / 30 kDa	ATMG00070.E	10	58.9	22.9	190	7297700
39 kDa	AT2G20360.1	23	66.7	37.7	344	6885100
PSST	AT5G11770.1	10	46.8	21.6	196	6849400
Cyt b	ATMG00220.E	4	10.9	44.2	392	6593500
QCR9	AT3G52730.1	5	31.9	8.4	72	6203300
NUXM	AT4G16450.2	6	64.2	11.3	106	5930100
ND7 / 49 kDa	ATMG00510.E	18	47.2	44.8	393	5888500
B13	AT5G52840.1	14	65.7	17.9	158	5722000
B17.2	AT3G03100.1	10	57.9	18.2	158	5718100
75 kDa	AT5G37510.1	37	60.5	74.8	686	5707300
B14.7	AT2G42210.4	7	64.8	17.0	159	5357900
B14.5b	AT4G20150.1	6	49.4	9.1	80	5106900
51 kDa	AT5G08530.1	24	56.4	50.1	456	4933400
18 kDa	AT5G67590.1	7	48.1	14.4	130	4452000
CAL1	AT5G63510.1	5	20.2	25.1	230	4246500
QCR10	AT2G40765.1	4	43.9	5.8	56	4209000
ND2	ATMG01320.E	6	11.4	55.5	499	4139400
FDX-CI	AT3G07480.1	9	60.4	14.5	132	4091200
Cyt c1-1	AT5G40810.2	6	33.1	27.2	243	3814700
Rieske-1	AT5G13430.1	11	40.1	23.2	212	3568600
QCR8-2	AT5G05370.1	2	26.4	8.3	71	3538300
CA3	AT5G66510.1	9	62.4	27.7	257	3534800
B15	AT2G31490.1	4	43.7	8.2	70	3380900
B12-2	AT2G02510.1	3	29.2	7.9	71	3139100
TYKY-1	AT1G79010.1	4	15.3	23.7	206	3083300
P2	AT2G27730.1	4	39.8	11.8	112	2926100
CAL2	AT3G48680.1	4	23.8	25.0	230	2899900
24 kDa	AT4G02580.1	9	40.8	24.6	222	2728400
B8	AT5G47890.1	4	48.5	10.7	96	2488800
PDSW-1	AT3G18410.2	3	27.4	12.3	105	2412400
ND1	ATMG01275.E	6	18.2	36.0	325	1875200
ND4	ATMG00580.E	5	10.9	55.9	494	1717800
B14	AT3G12260.1	8	74.4	15.0	132	1667700
CA1	AT1G19580.1	13	53.1	29.8	274	1655200
B9	AT2G46540.1	2	36.9	6.7	64	1652400
PDSW-2	AT1G49140.1	4	35.5	12.4	106	1583800
MPP-α-2	AT3G16480.1	10	24	51.1	473	1493700
P1 (SGDH)	AT1G67350.2	8	82.7	11.7	97	1485900
15 kDa-1	AT3G62790.1	2	25.3	9.8	82	1404300
B18	AT2G02050.1	6	61.2	11.7	103	1276900
ND5	ATMG00513.E	10	17.2	74.5	669	1270900
QCR6-1 (Hinge)	AT1G15120.1	6	68.1	7.9	68	1047100
AGGG	AT1G76200.1	1	14.5	7.4	68	1037000
MWFE	AT3G08610.1	1	20	7.2	64	1035500
KFYI	AT4G00585.1	6	64.8	9.7	87	850830
TYKY-2	AT1G16700.1	3	13.5	23.6	206	827880
ND3	ATMG00990.E	1	12.6	13.9	119	825730
B16.6-1	AT1G04630.1	2	9.8	16.0	142	744950
ND6	ATMG00270.E	2	6.8	23.7	205	740400
ND4L	ATMG00650.E	1	13	11.2	100	609300
ASHI	AT5G47570.1	2	13.6	10.2	95	271760
ESSS-2	AT3G57785.1	3	20.2	10.1	91	231000
PGIV-2	AT5G18800.2	2	30.2	11.8	105	186910
QCR7-2 (14 kDa)	AT5G25450.1	3	33.6	14.5	121	176920
13 kDa	AT3G03070.1	1	11.8	9.7	88	169770
P9	AT1G67785.1	1	19	7.4	62	142960
SDAP-1	AT1G65290.1	1	14.3	14.2	126	140490
SDAP-2	AT2G44620.1	3	23	13.7	122	108310
PGIV-1	AT3G06310.1	1	23.1	12.2	108	16910

Supplementary Table 4: Subunits of *Arabidopsis* I+III$_2$ supercomplex identified by mass spectrometry. Column 1: names of subunits in blue for complex I / in red for complex III$_2$ (see Supplementary Tables 5 and 6 for nomenclatures of complex I and complex III$_2$ subunits in different species); Column 2: Accession numbers (TAIR, https://www.arabidopsis.org/); column 3: number of unique peptides; column 4: sequence coverage [%]; column 5: calculated molecular mass of the mature proteins [kDa]. Column 6: Number of amino acids of the mature proteins. Column 7: Intensity-based absolute quantification (iBAQ) score.

A. thaliana	accession	*C. reinhardtii*	accession	bovine	human	*Yarrowia*	*E. coli*	*T. therm.*
**► *Peripheral arm (incl. bridge domain)***								
-		10 kDa	Cre03.g178250	10 kDa	NDUFV3	-	-	-
13 kDa	At3g03070	13 kDa	Cre03.g146247	13 kDa	NDUFS6	NUMM	-	-
18 kDa	At5g67590	18 kDa	Cre10.g450400	18 kDa	NDUFS4	NUYM	-	-
24 kDa	At4g02580	24 kDa	Cre10.g434450	24 kDa	NDUFV2	NUHM	NUOE	NQO2
39 kDa	At2g20360	39 kDa	Cre10.g422600	39 kDa	NDUFA9	NUEM	-	-
51 kDa	At5g08530	51 kDa	Cre12.g535950	51 kDa	NDUFV1	NUBM	NUOF	NQO1
75 kDa	At5g37510	75 kDa	Cre16.g679500	75kDa	NDUFS10	NUAM	NUOG	NQO3
B8	At5g47890	B8	Cre13.g568800	B8	NDUFA2	NI8M	-	-
B13	At5g52840	B13	Cre12.g484700	B13	NDUFA5	NUFM	-	-
B14	At3g12260	B14	Cre12.g555250	B14	NDUFA6	NB4M	-	-
B14.5a	At5g08060	B14.5a	Cre11.g467767	B14.5a	NDUFA7	NUZM	-	-
B17.2	At3g03100	B17.2	Cre09.g405850	B17.2	NDUFA12	N7BM	-	-
ND7 (49 kDa)	AtMg00510	ND7	Cre07.g327400	49 kDa	NDUFS2	NUCM	NUOD	NQO3
ND9 (30 kDa)	AtMg00070	ND9	Cre12.g492300	30 kDa	NDUFS3	NUGM	NUOC	NQO5
PSST	At5g11770	PSST	-	PSST	NDUFS7	NUKM	NUOB	NQO6
SDAP-2 (mtACP-2)	At2g44620	SDAP1/2	Cre16.g673109	SDAP-β	NDUFAB1	ACPM2	-	-
TYKY-1	At1g79010	TYKY	Cre12.g496750	TYKY	NDUFS8	NUIM	NUOI	NQO9
TYKY-2	At1g16700							
C1-Ferredoxin	At3g07480	NUOP3	Cre02.g100200	-	-	-	-	-
-		-		-	-	-	-	NQO15
**► *Membrane arm (incl. carbonic anhydrase domain)***								
15 kDa-1	At3g62790	15 kDa	Cre12.g511200	15kDa	NDUFS5	NIPM	-	-
15 kDa-2	At2g47690							
-		-		42 kDa	NDUFA10	-	-	-
AGGG	At1g76200	AGGG	n.d.	AGGG	NDUFB2	NIGM	-	-
ASHI	At5g47570	ASHI	Cre01.g007850	ASHI	NDUFB8	NIAM	-	-
B9	At2g46540	B9	Cre12.g537050	B9	NDUFA3	NI9M	-	-
B12-1	At1g14450	B12	Cre05.g244850	B12	NDUFB3	NB2M	-	-
B12-2	At2g02510							
B14.5b	At4g20150	B14.5b	Cre13.g571150	B14.5b	NDUFC2	N4BM	-	-
B14.7	At2g42210	B14.7	Cre14.g617826	B14.7	NDUFA11	NUJM	-	-
B15	At2g31490	B15	Cre03.g204650	B15	NDUFB4	NB5M	-	-
B16.6-1	At1g04630	B16.6	Cre16.g664600	B16.6	GRIM19	NB6M	-	-
B16.6-2	At2g33220				= NDUFA13			
-		-		B17	NDUFB6	-	-	-
B18	At2g02050	B18	Cre06.g278188	B18	NDUFB7	NB8M	-	-
B22	At4g34700	B22	Cre11.g467668	B22	NDUFB9	NI2M	-	-
ESSS-1	At2g42310	ESSS	Cre05.g240800	ESSS	NDUFB11	NESM	-	-
ESSS-2	At3g57785							
KFYI	At4g00585	KFYI	Cre17.g725400	KFYI	NDUFC1	-	-	-
				MNLL	NDUFB1	-	-	-
MWFE	At3g08610	MWFE	Cre10.g459750	MWFE	NDUFA1	NIMM	-	-
ND1	AtMg00516	ND1	AAB93446	ND1	ND1	NU1M	NUOH	NQO8
	AtMg01120							
	AtMg01275							
ND2	AtMg00285	ND2	AAB93444	ND2	ND2	NU2M	NUON	NQO14
	AtMg01320							
ND3	AtMg00990	ND3	Cre08.g378900	ND3	ND3	NU3M	NUOA	NQO7
ND4	AtMg00580	ND4	AAB93441	ND4	ND4	NU4M	NUOM	NQO13
ND4L	AtMg00650	ND4L	Cre09.g402552	ND4L	ND4L	NULM	NUOK	NQO11
ND5	AtMg00060	ND5	AAB93442	ND5	ND5	NU5M	NUOL	NQO12
	AtMg00513							
	AtMg00665							
ND6	AtMg00270	ND6	AAB93445	ND6	ND6	NU6M	NUOJ	NQO10
PDSW-1	At3g18410	PDSW	Cre12.g555150	PDSW	NDUFB10	NIDM	-	-
PDSW-2	At1g49140							
PGIV-1	At3g06310	PGIV	Cre07.g333900	PGIV	NDUFA8	NUPM	-	-
PGIV-2	At5g18800							
SDAP-1 (mtACP-1)	At1g65290	SDAP1/2	Cre16.g673109	SDAP-α	NDUFAB1	ACPM1	-	-
NUXM	At4g16450	NUXM	Cre06.g267200	-	-	NUXM	-	-
P1 (SGDH?)	At1g67350	-		SGDH	NDUFB5	NUNM	-	-
P2	At2g27730	-		-	-	-	-	-
P9	At1g67785	NUOP9	Cre03.g178250	-	-	-	-	-
-		NUOP4	Cre08.g378550	-	-	-	-	-
-		NUOP5	Cre08.g378050	-	-	-	-	-
-		NUOP7	XP_001698808	-	-	-	-	-
-		NUOP8	XP_001692071	-	-	-	-	-
CA2	At1g47260	CA2	Cre09.g415850	-	-	-	-	-
CA1	At1g19580	CA1/CA3	Cre12.g516450	-	-	-	-	-
CA3	At5g66510			-	-	-	-	-
CAL1	At5g63510	CAL	Cre06.g293850	-	-	-	-	-
CAL2	At3g48680			-	-	-	-	-
-		-		-	-	NUUM	-	-
-		-		-	-	ST1	-	-

Supplementary Table 5: Nomenclature of complex I subunits in *Arabidopsis* and other model species. Extensions -1 and -2 indicate isoforms present in *Arabidopsis*. In our atomic models of the I+III_2 supercomplex (Fig. 1, Fig. 2), we used the isoform which is more abundant according to our MS data (Supplementary Table 4). Subunits specific for clades are highlighted by colors.

A. thaliana	accession	bovine	human	yeast	*R. capsulatus*
MPP-α-1<br>MPP-α-2	At1g51980<br>At3g16480	Core 2	UQCRC2	Core 2	-
MPP-β	At3g02090	Core 1	UQCRC1	Core 1	-
Cyt b	AtMg00220	Cyt b	MT-CYB	Cyt b	Cyt b
Cyt c1-1<br>Cyt c1-2	At5g40810<br>At3g27240	Cyt c1	CYT1	Cyt c1	Cyt c1
Rieske-1<br>Rieske-2	At5g13430<br>At5g13440	Rieske	UQCRFS1	Rieske	Rieske
QCR6-1 (Hinge)<br>QCR6-2 (Hinge)	At1g15120<br>At2g01090	Hinge<br>(11 kDa)	UQCRH	QCR6	
QCR7-1 (14 kDa)<br>QCR7-2 (14 kDa)	At4g32470<br>At5g25450	14 kDa	UQCRB	QCR7	-
QCR8-1<br>QCR8-2	At3g10860<br>At5g05370	9.5 kDa	UQCRQ	QCR8	-
QCR9	At3g52730	7.2 kDa	UQCR10	QCR9	-
QCR10	At2g40765	6.4 kDa	UQCR11	QCR10	-

Supplementary Table 6: Nomenclature of complex III subunits in *Arabidopsis* and other model species. Extensions -1 and -2 indicate isoforms present in *Arabidopsis*. In our atomic models of the I+III_2 supercomplex (Fig. 1, Fig. 2), we used the isoform which is more abundant according to our MS data (MPP-α-1, Cyt c1-1, Rieske-1, QCR6-1, QCR7-1, QCR8-2; see Supplementary Table 4).

Name	Accession	precursor length (no. of amino acids)	preseq. length (no of amino acids)	mature sequence length (no of amino acids)	mature mass (kDa)	mature IEP	mature GRAVY
**13 kDa**	At3g03070	110	22	88	9.7	5.6	-0.306
**15 kDa-1**	At3g62790	83	1	82	9.8	7.9	-1.288
**18 kDa**	At5g67590	154	24	130	14.4	9.1	-0.770
**24 kDa**	At4g02580	255	33	222	24.6	5.9	-0.402
**39 kDa**	At2g20360	402	58	344	37.7	7.9	-0.028
**51 kDa**	At5g08530	486	30	456	50.1	7.5	-0.329
**75kDa**	At5g37510	745	59	686	74.8	6.0	-0.141
**AGGG**	At1g76200	69	1	68	7.4	9.0	-0.572
**ASHI**	At5g47570	125	30	95	10.2	4.7	0.057
**B8**	At5g47890	97	1	96	10.7	9.5	-0.311
**B9**	At2g46540	65	1	64	6.7	9.1	0.530
**B12-2**	At2g02510	72	1	71	7.9	10.0	-0.537
**B13**	At5g52840	169	11	158	17.9	4.6	-0.583
**B14**	At3g12260	133	1	132	15.0	9.1	-0.255
**B14.5a**	At5g08060	131	1	130	14.9	8.6	-0.734
**B14.5b**	At4g20150	81	1	80	9.1	8.7	-0.226
**B14.7**	At2g42210	159	0	159	17.0	7.9	-0.050
**B15**	At2g31490	71	1	70	8.2	9.3	-0.534
**B16.6-2**	At2g33220	143	1	142	16.0	9.36	-0.489
**B17.2**	At3g03100	159	1	158	18.2	9.3	-0.792
**B18**	At2g02050	103	0	103	11.7	7.7	-0.428
**B22**	At4g34700	117	1	116	13.5	8.7	-0.748
**CA1**	At1g19580	275	1	274	29.8	7.3	-0.231
**CA2**	At1g47260	278	1	277	29.9	6.8	-0.165
**CAL2**	At3g48680	256	26	230	25.0	6.4	0.035
**ESSS-1**	At2g42310	114	23	91	10.1	5.6	-0.598
**FDX-CI**	At3g07480	159	27	132	14.5	5.6	-0.323
**KFYI**	At4g00585	88	1	87	9.7	9.5	-0.621
**MWFE**	At3g08610	65	1	64	7.2	7.9	-0.222
**ND1**	AtMg00516 / AtMg01120 / AtMg01275	325	0	325	36.0	8.3	0.987
**ND2**	AtMg00285 / AtMg01320	499	0	499	55.5	8.7	0.936
**ND3**	AtMg00990	119	0	119	13.9	4.4	0.892
**ND4**	AtMg00580	495	1	494	55.9	8.8	0.949
**ND4L**	AtMg00650	100	0	100	11.2	5.0	1.394
**ND5**	AtMg00060 / AtMg00513 / AtMg00665	669	0	669	74.5	6.6	0.753
**ND6**	AtMg00270	205	0	205	23.7	9.6	0.876
**ND7 / 49 kDa**	AtMg00510	394	1	393	44.8	6.7	-0.181
**ND9 / 30 kDa**	AtMg00070	190	0	190	22.9	6.9	-0.581
**NUXM**	At4g16450	106	0	106	11.3	9.9	0.024
**P1**	At1g67350	98	1	97	11.7	5.35	-1.010
**P2**	At2g27730	113	1	112	11.8	9.6	-0.488
**P9**	At1g67785	63	1	62	7.4	6.9	-0.455
**PDSW-1**	At3g18410	106	1	105	12.3	8.0	-0.983
**PGIV-2**	At5g18800	106	1	105	11.8	7.49	-0.514
**PSST**	At5g11770	218	22	196	21.6	9.2	-0.160
**SDAP-1**	At1g65290	126	0	126	14.2	4.6	-0.194
**SDAP-2**	At2g44620	122	0	122	13.7	4.9	-0.148
**TYKY-1**	At1g79010	222	16	206	23.7	5.0	-0.599
**Σ**		9,440	404	**9,036**	**1009.7**		
**Ø**		197		188.25	21.0	7.5	-0.199

Supplementary Table 7: Calculated properties of the 48 complex I subunits of *Arabidopsis*. The molecular mass refers to the mass of the mature subunits (excluding mitochondrial targeting sequences, if present, or removed initiator methionines; data from[2]. For subunits occurring in isoforms, the predominant isoform within our Cryo-EM structure (Fig. 1, Fig. 2) was selected, respectively. Note that the molecular mass calculation does not include the prosthetic groups (see Extended Data Fig. 2) attached to several of the subunits. Some residues appear at a higher mass due to posttranslational modifications.

Arabidopsis complex I includes 48 subunits with overall 9,036 amino acids; it has a molecular mass of 1,009.7 kDa.

Name	Accession	precursor length (no. of amino acids)	preseq. length (no of amino acids)	mature sequence length (no of amino acids)	mature mass (kDa)	mature IEP	mature GRAVY
MPP-α-1	At1g51980	503	26	477	51.5	5.29	-0.11
MPP-β	At3g02090	531	25	506	56.2	5.74	-0.34
Cyt b	AtMg00220	393	1	392	44.2	6.64	0.63
Cyt c1-1	At5g40810	307	64	243	27.2	5.12	-0.37
Rieske-1	At5g13430	272	60	212	23.2	6.51	-0.12
QCR6-1	At1g15120	69	1	68	7.9	7.78	-0.67
QCR7-1	At4g32470	122	1	121	14.4	9.73	-0.65
QCR8-2	At5g05370	72	1	71	8.3	9.48	-0.28
QCR9	At3g52730	72	0	72	8.4	9.45	-0.47
QCR10	At2g40765	57	1	56	5.8	8.42	0.20
Σ		2,398	180	**2,218**	**247.1**		
ø		240		221,8	24.7	7.4	-0.218

Supplementary Table 8: Calculated properties of the 10 complex III subunits in *Arabidopsis*. The molecular mass refers to the mature subunits (excluding mitochondrial targeting sequences, if present, or removed initiator methionines; data in accordance with *Solanum tuberosum*[3]. For subunits present in different isoforms, the predominant isoform within our cryo-EM structure (Fig. 1, Fig. 2) is selected, respectively. Note that the molecular mass calculation does not include prosthetic groups (see Extended Data Fig. 2) attached to some subunits. Some residues appear at a higher mass due to posttranslational modifications.

Arabidopsis complex III_2 includes 2x10 subunits with overall 4,436 amino acids; it has a molecular mass of 494.2 kDa.

The *Arabidopsis* I+III_2 supercomplex includes 48 complex I subunits and 2x10 complex III subunits = 68 subunits. The supercomplex includes 13,472 amino acids. It has a calculated molecular mass of 1,503.9 kDa

Arabidopsis complex III_2			*Arabidopsis* complex I	
Amino acid	copy number		Amino acid	copy number
L	432		L	895
A	408		A	700
V	326		G	683
S	316		V	657
G	296		S	597
E	266		I	573
P	252		E	529
I	242		R	490
K	228		K	473
D	222		F	471
T	220		P	465
R	216		T	440
Y	194		D	422
F	190		Y	310
N	156		N	304
Q	138		M	268
H	112		Q	253
M	108		H	194
W	70		C	164
C	44		W	148
∑	4436		∑	9036

Supplementary Table 9: Amino acid composition of *Arabidopsis* complex III_2 (2x10 subunits) and *Arabidopsis* complex I (48 subunits).

References for Supplementary Figures and Tables

1. Dudkina, N.V., Eubel, H., Keegstra, W., Boekema, E.J. & Braun, H.P. Structure of a mitochondrial supercomplex formed by respiratory-chain complexes I and III. *Proc Natl Acad Sci U S A* **102**, 3225-3229 (2005).
2. Senkler, J., Senkler, M. & Braun, H.P. Structure and function of complex I in animals and plants - a comparative view. *Physiologia Plantarum* **161**, 6-15 (2017).
3. Braun, H.P. & Schmitz, U.K. The bifunctional cytochrome c reductase/processing peptidase complex from plant mitochondria. *J Bioenerg Biomembr* **27**, 423-36 (1995).
