# Supplementary material for: Cryo-EM structure of the respiratory I + III2 supercomplex from Arabidopsis thaliana at 2 Å resolution
Source: Nat Plants. 2022 Dec 30;9(1):142–56. doi: 10.1038/s41477-022-01308-6 (PMC9873573; doi:10.1038/s41477-022-01308-6)
Supplement: Supplementary file 2 — Reporting Summary [file 41477_2022_1308_MOESM2_ESM.pdf]

nature portfolio

Corresponding author(s): Hans-Peter Braun
Niklas Klusch

Last updated by author(s): Dec 12, 2022

Reporting Summary

Nature Portfolio wishes to improve the reproducibility of the work that we publish. This form provides structure for consistency and transparency in reporting. For further information on Nature Portfolio policies, see our Editorial Policies and the Editorial Policy Checklist.

Statistics

For all statistical analyses, confirm that the following items are present in the figure legend, table legend, main text, or Methods section.

n/a	Confirmed	
☐	☒	The exact sample size (*n*) for each experimental group/condition, given as a discrete number and unit of measurement
☐	☒	A statement on whether measurements were taken from distinct samples or whether the same sample was measured repeatedly
☒	☐	The statistical test(s) used AND whether they are one- or two-sided<br>*Only common tests should be described solely by name; describe more complex techniques in the Methods section.*
☒	☐	A description of all covariates tested
☒	☐	A description of any assumptions or corrections, such as tests of normality and adjustment for multiple comparisons
☒	☐	A full description of the statistical parameters including central tendency (e.g. means) or other basic estimates (e.g. regression coefficient) AND variation (e.g. standard deviation) or associated estimates of uncertainty (e.g. confidence intervals)
☒	☐	For null hypothesis testing, the test statistic (e.g. *F*, *t*, *r*) with confidence intervals, effect sizes, degrees of freedom and *P* value noted<br>*Give P values as exact values whenever suitable.*
☒	☐	For Bayesian analysis, information on the choice of priors and Markov chain Monte Carlo settings
☒	☐	For hierarchical and complex designs, identification of the appropriate level for tests and full reporting of outcomes
☒	☐	Estimates of effect sizes (e.g. Cohen's *d*, Pearson's *r*), indicating how they were calculated

Our web collection on statistics for biologists contains articles on many of the points above.

Software and code

Policy information about availability of computer code

Data collection	TimsTOF operating software HyStar 6.0.30.0 (Bruker) was used for the acquisition of mass spectrometry proteomics data.<br><br>Cryo-EM data were collected with a with a Titan Krios G4i (Thermo Scientific) at 300 kV and a nominal magnification of 215,000x, corresponding to a pixel size of 0.573 Å. The microscope was equipped with a cold field emission gun and a Falcon 4 camera (Thermo Scientific) operating in electron counting mode. EER Movies consisting of 1,118 raw frames were recorded automatically at an exposure rate of 3.4 e-/pix/s and a total dose of 50 e-/Å2 using the EPU software (Thermo Scientific).
Data analysis	Analyses of mass spectrometry proteomics data were carried out with MaxQuant, Version 2.0.3.0.<br><br>Cryo-EM movies were motion-corrected using MotionCor2 and CTF was estimated with CTFFind4.1.13. Particles were picked with crYOLO and imported into Relion3.1.3 for further processing. Final B-factor sharpened cryo-EM densities were further modified with the phenix.resolve_cryo_em tool. Atomic models were build manually with Coot based on previously published models of complex I from Arabidopsis thaliana (pdb:7ARB) and homology models for each individual subunit of complex III created by the SWISS-MODEL server. Water molecules were built using the Segger Chimera SWIM tool. Aqueous cavities were calculated with Hollow.

For manuscripts utilizing custom algorithms or software that are central to the research but not yet described in published literature, software must be made available to editors and reviewers. We strongly encourage code deposition in a community repository (e.g. GitHub). See the Nature Portfolio guidelines for submitting code & software for further information.

Data

Policy information about availability of data

All manuscripts must include a data availability statement. This statement should provide the following information, where applicable:

- Accession codes, unique identifiers, or web links for publicly available datasets
- A description of any restrictions on data availability
- For clinical datasets or third party data, please ensure that the statement adheres to our policy

The mass spectrometry proteomics data of the mitochondrial I+III2 supercomplex from Arabidopsis thaliana are available at the PRIDE repository (https://www.ebi.ac.uk/pride/), dataset identifier: PXD036482

Cryo-EM density maps and atomic models of the I+III2 supercomplex from Arabidopsis thaliana are available at the Electron microscopy (EMDB, https://www.ebi.ac.uk/emdb/, accessions EMD-15998, EMD-15999, EMD-16000, EMD-16003, EMD-16007, EMD-16008, EMD-16168, EMD-16171, EMD-16172) and Protein Data Bank (PDB, https://www.rcsb.org/, accessions 8BED, 8BEE, 8BEF, 8BEH, 8BEL, 8BEP, 8BPX, 8BQ5, 8BQ6). Details are given in Supplemental Tables 2 and 3.

Human research participants

Policy information about studies involving human research participants and Sex and Gender in Research.

Reporting on sex and gender	*Use the terms sex (biological attribute) and gender (shaped by social and cultural circumstances) carefully in order to avoid confusing both terms. Indicate if findings apply to only one sex or gender; describe whether sex and gender were considered in study design whether sex and/or gender was determined based on self-reporting or assigned and methods used. Provide in the source data disaggregated sex and gender data where this information has been collected, and consent has been obtained for sharing of individual-level data; provide overall numbers in this Reporting Summary. Please state if this information has not been collected. Report sex- and gender-based analyses where performed, justify reasons for lack of sex- and gender-based analysis.*
Population characteristics	*Describe the covariate-relevant population characteristics of the human research participants (e.g. age, genotypic information, past and current diagnosis and treatment categories). If you filled out the behavioural & social sciences study design questions and have nothing to add here, write "See above."*
Recruitment	*Describe how participants were recruited. Outline any potential self-selection bias or other biases that may be present and how these are likely to impact results.*
Ethics oversight	*Identify the organization(s) that approved the study protocol.*

Note that full information on the approval of the study protocol must also be provided in the manuscript.

Field-specific reporting

Please select the one below that is the best fit for your research. If you are not sure, read the appropriate sections before making your selection.

☒ Life sciences ☐ Behavioural & social sciences ☐ Ecological, evolutionary & environmental sciences

For a reference copy of the document with all sections, see nature.com/documents/nr-reporting-summary-flat.pdf

Life sciences study design

All studies must disclose on these points even when the disclosure is negative.

Sample size	Four idenpendant biochemical purifications of the I+III2 supercomplex from Arabidopsis thaliana were carried out. All fractions were biochemically and structurally analysed.
Data exclusions	No data were excluded.
Replication	Arabidopsis thaliana I+III2 supercomplex cryo-EM data were collected 4 times for the analysis. All attempts at replication were successful.
Randomization	All plants were grown under identical conditions in a climate chamber.
Blinding	Cryo-EM datasets were processed independently before being combined.

Reporting for specific materials, systems and methods

We require information from authors about some types of materials, experimental systems and methods used in many studies. Here, indicate whether each material, system or method listed is relevant to your study. If you are not sure if a list item applies to your research, read the appropriate section before selecting a response.

Materials & experimental systems

n/a	Involved in the study
☒	☐ Antibodies
☐	☒ Eukaryotic cell lines
☒	☐ Palaeontology and archaeology
☒	☐ Animals and other organisms
☒	☐ Clinical data
☒	☐ Dual use research of concern

Methods

n/a	Involved in the study
☒	☐ ChIP-seq
☒	☐ Flow cytometry
☒	☐ MRI-based neuroimaging

Eukaryotic cell lines

Policy information about cell lines and Sex and Gender in Research

Cell line source(s)	An Arabidopsis thaliana cell line has been established from leaves
Authentication	*Describe the authentication procedures for each cell line used OR declare that none of the cell lines used were authenticated.*
Mycoplasma contamination	*Confirm that all cell lines tested negative for mycoplasma contamination OR describe the results of the testing for mycoplasma contamination OR declare that the cell lines were not tested for mycoplasma contamination.*
Commonly misidentified lines (See ICLAC register)	*Name any commonly misidentified cell lines used in the study and provide a rationale for their use.*
